# Supplementary figures and images for: The spinal cord facilitates cerebellar upper limb motor learning and control; inputs from neuromusculoskeletal simulation
Source: PLoS Comput Biol. 2024 Jan 2;20(1):e1011008. doi: 10.1371/journal.pcbi.1011008 (PMC10786408; doi:10.1371/journal.pcbi.1011008)

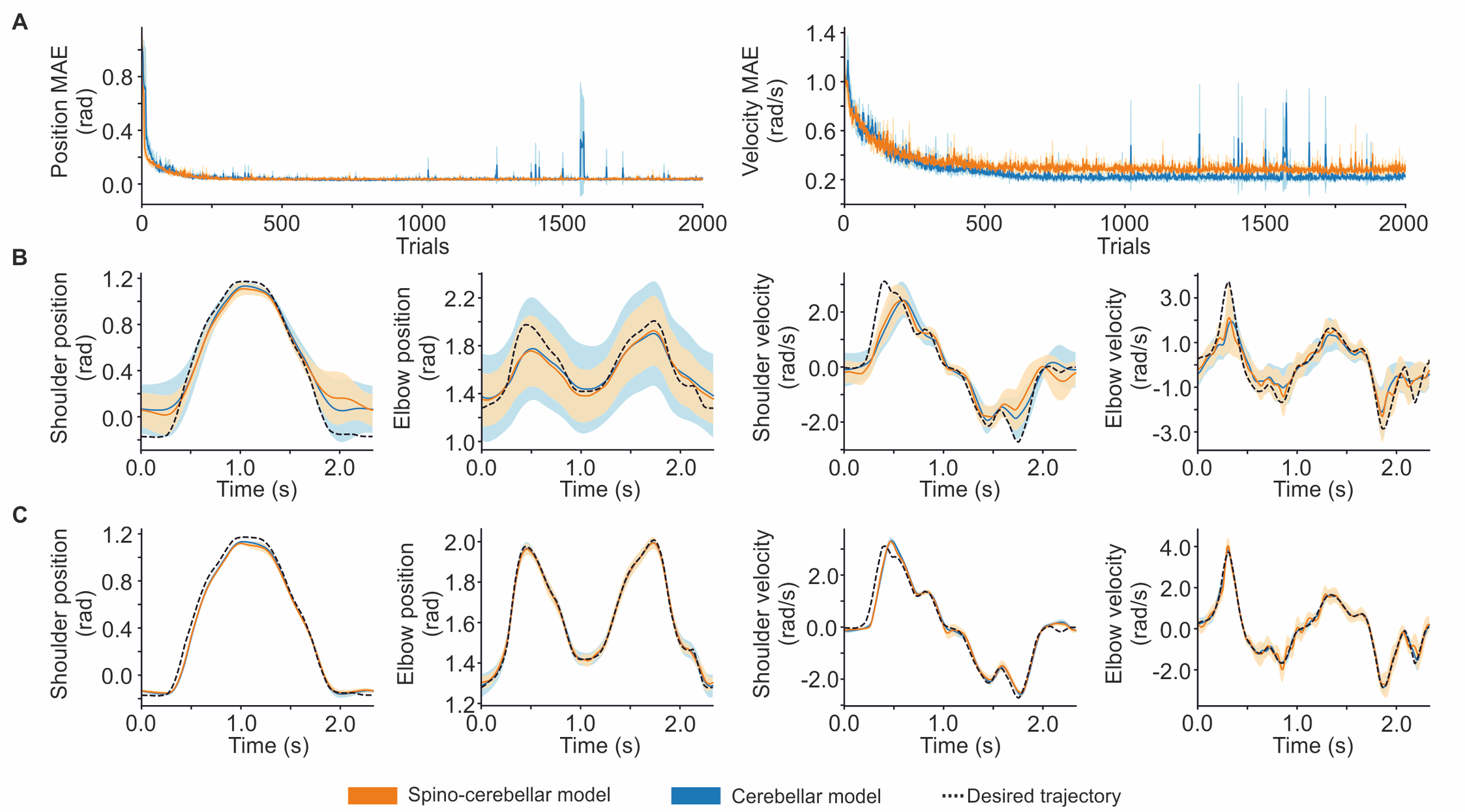

Supplement: S1 Fig — A) Position and velocity mean absolute error (MAE) over the 2000-trial motor adaptation process for both the spino-cerebellar and cerebellar models performing P1’s slow flexion-extension (2.3 s). B) Joint kinematics of the first 200 trials (mean and standard deviation, std) for both models performing P1’s slow flexion-extension (2.3 s). C) Joint kinematics of the last 200 trials (mean and std) for both models performing P1’s slow flexion-extension (2.3 s). (TIF) [file pcbi.1011008.s001.tif]

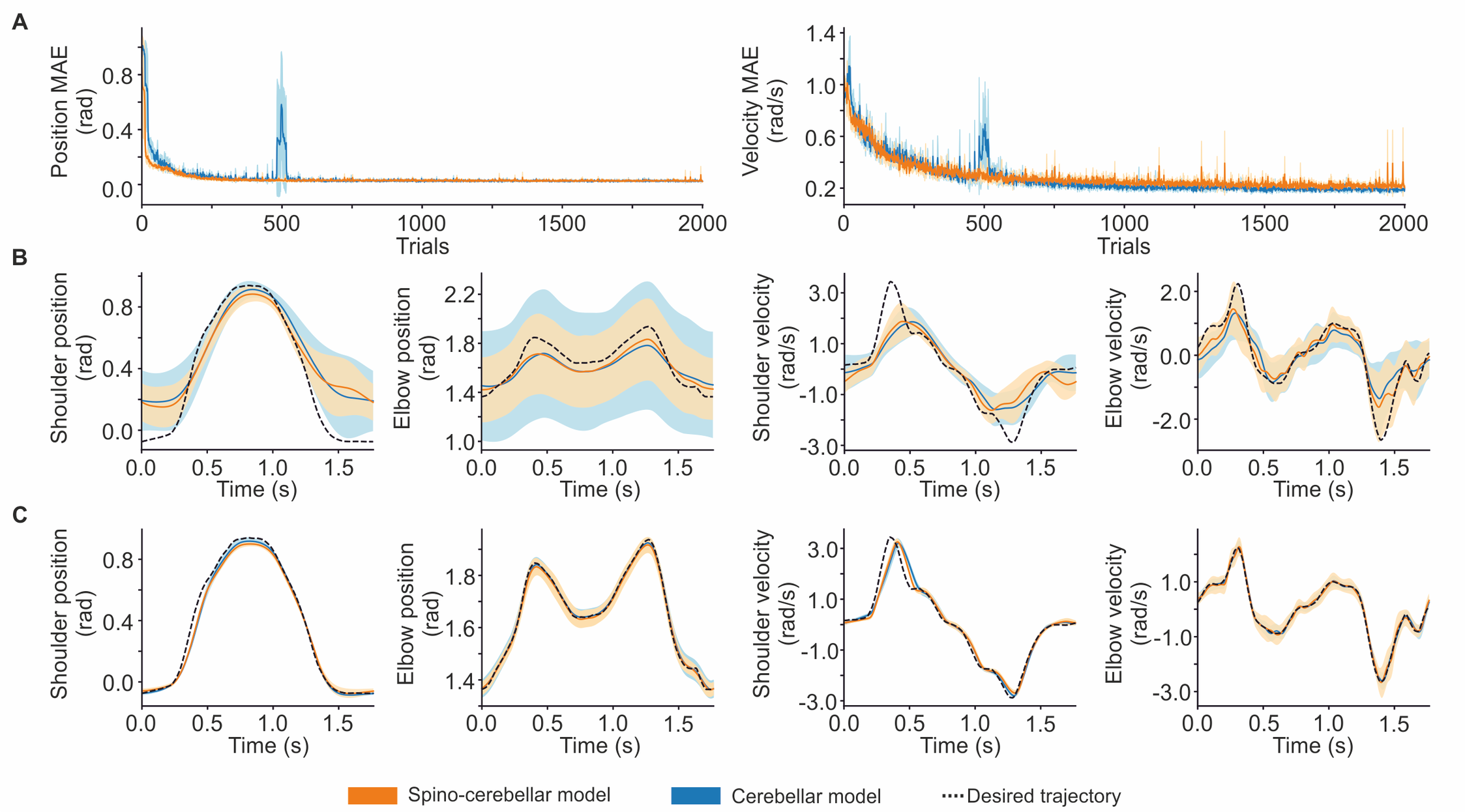

Supplement: S2 Fig — A) Position and velocity mean absolute error (MAE) over the 2000-trial motor adaptation process for both the spino-cerebellar and cerebellar models performing P1’s moderate flexion-extension (1.7 s). B) Joint kinematics of the first 200 trials (mean and standard deviation, std) for both models performing P1’s moderate flexion-extension (1.7 s). C) Joint kinematics of the last 200 trials (mean and std) for both models performing P1’s moderate flexion-extension (1.7 s). (TIF) [file pcbi.1011008.s002.tif]

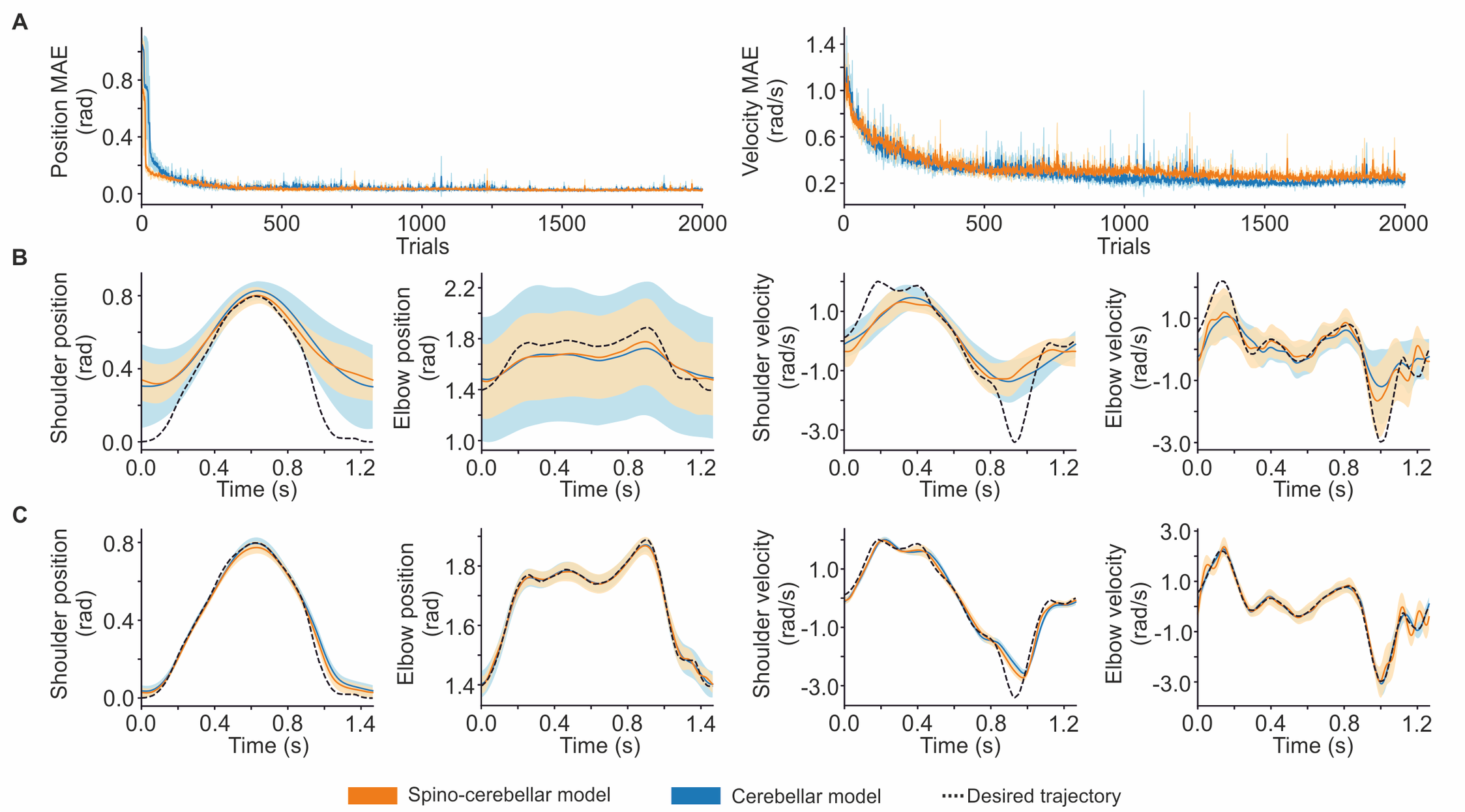

Supplement: S3 Fig — A) Position and velocity mean absolute error (MAE) over the 2000-trial motor adaptation process for both the spino-cerebellar and cerebellar models performing P1’s fast flexion-extension (1.3 s). B) Joint kinematics of the first 200 trials (mean and standard deviation, std) for both models performing P1’s fast flexion-extension (1.3 s). C) Joint kinematics of the last 200 trials (mean and std) for both models performing P1’s fast flexion-extension (1.3 s). (TIF) [file pcbi.1011008.s003.tif]

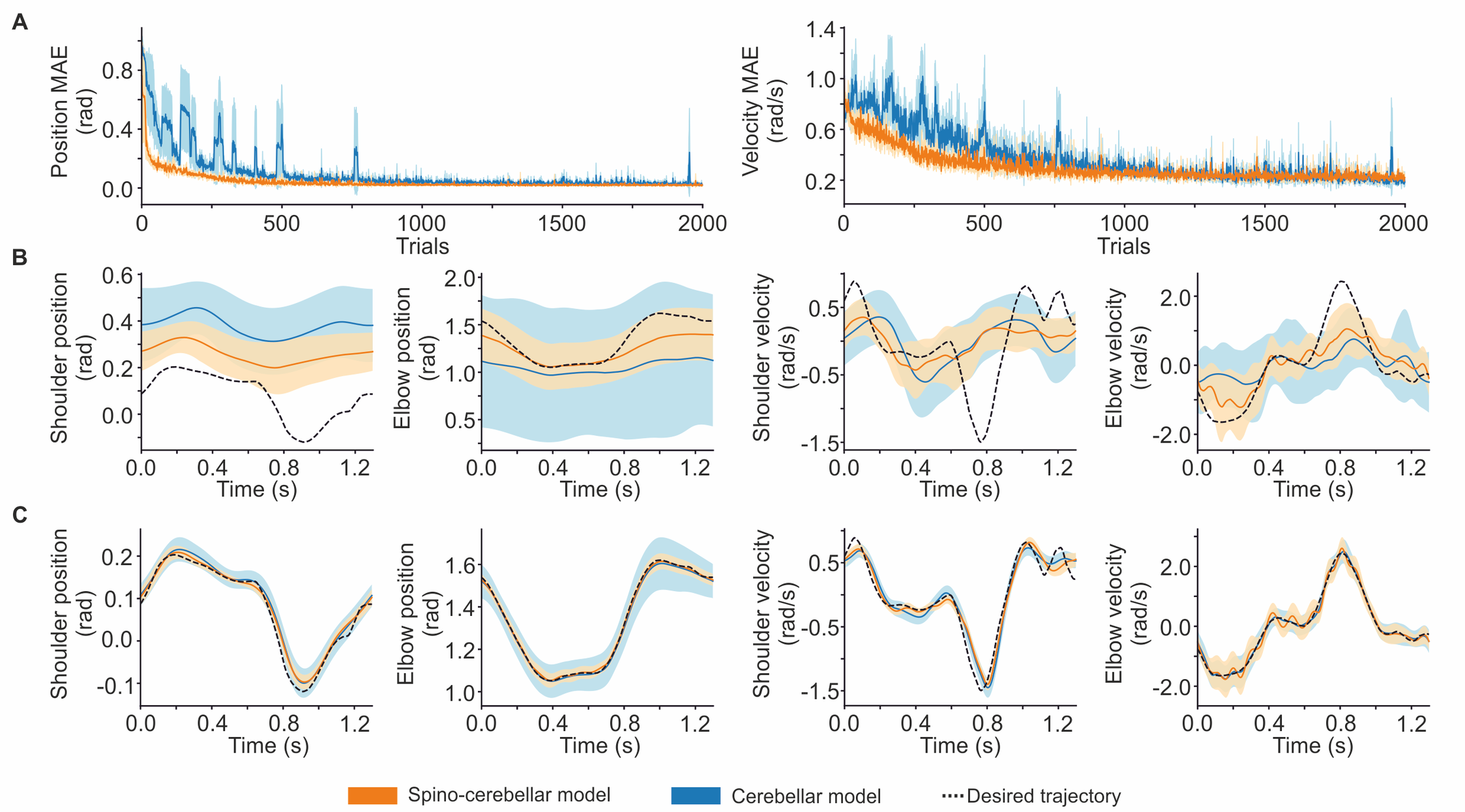

Supplement: S4 Fig — A) Position and velocity mean absolute error (MAE) over the 2000-trial motor adaptation process for both the spino-cerebellar and cerebellar models performing P1’s moderate circle trajectory (1.3 s). B) Joint kinematics of the first 200 trials (mean and standard deviation, std) for both models performing P1’s moderate circle trajectory (1.3 s). C) Joint kinematics of the last 200 trials (mean and std) for both models performing P1’s moderate circle trajectory (1.3 s). (TIF) [file pcbi.1011008.s004.tif]

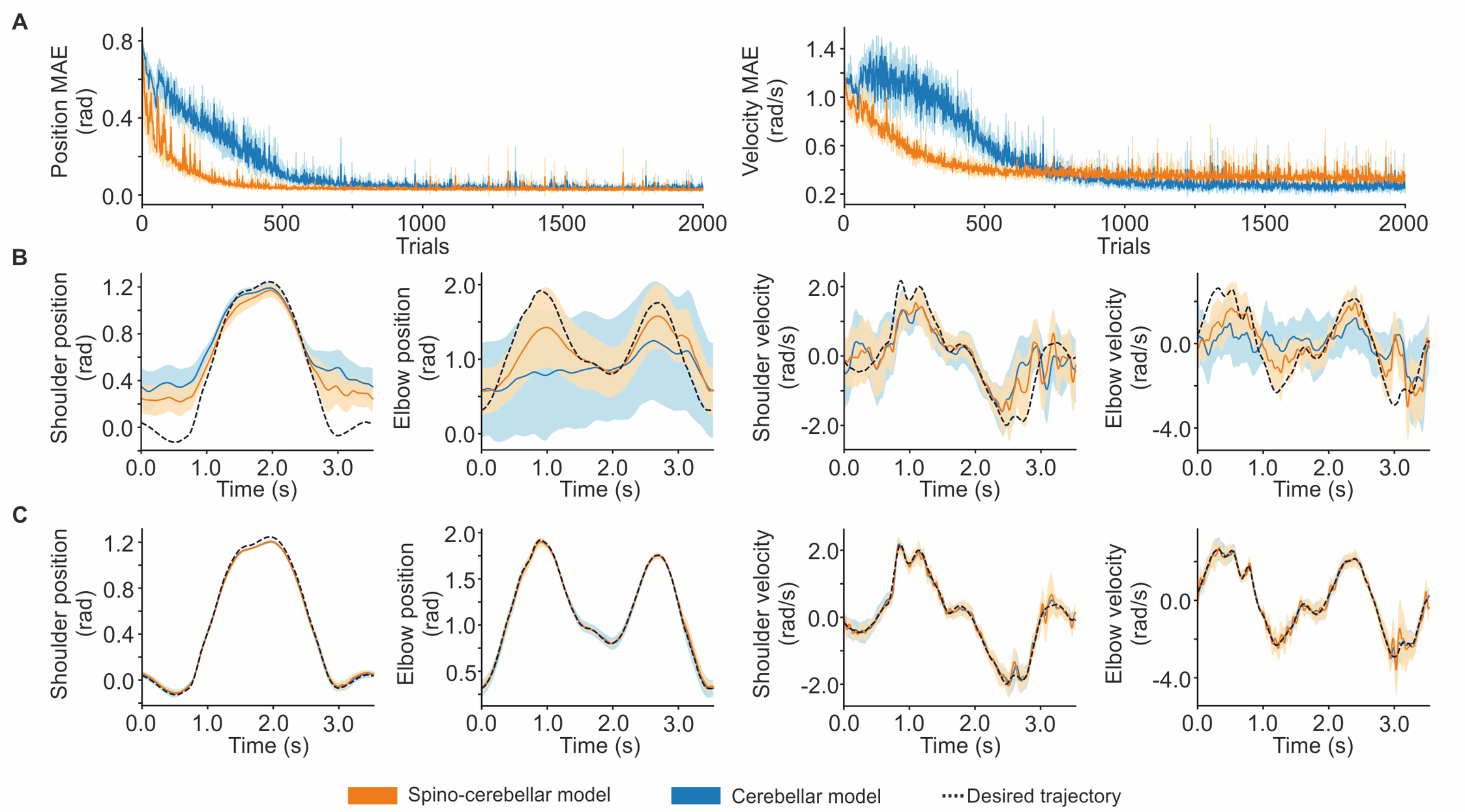

Supplement: S5 Fig — A) Position and velocity mean absolute error (MAE) over the 2000-trial motor adaptation process for both the spino-cerebellar and cerebellar models performing P2’s slow flexion-extension (3.5 s). B) Joint kinematics of the first 200 trials (mean and standard deviation, std) for both models performing P2’s slow flexion-extension (3.5 s). C) Joint kinematics of the last 200 trials (mean and std) for both models performing P2’s slow flexion-extension (3.5 s). (TIF) [file pcbi.1011008.s005.tif]

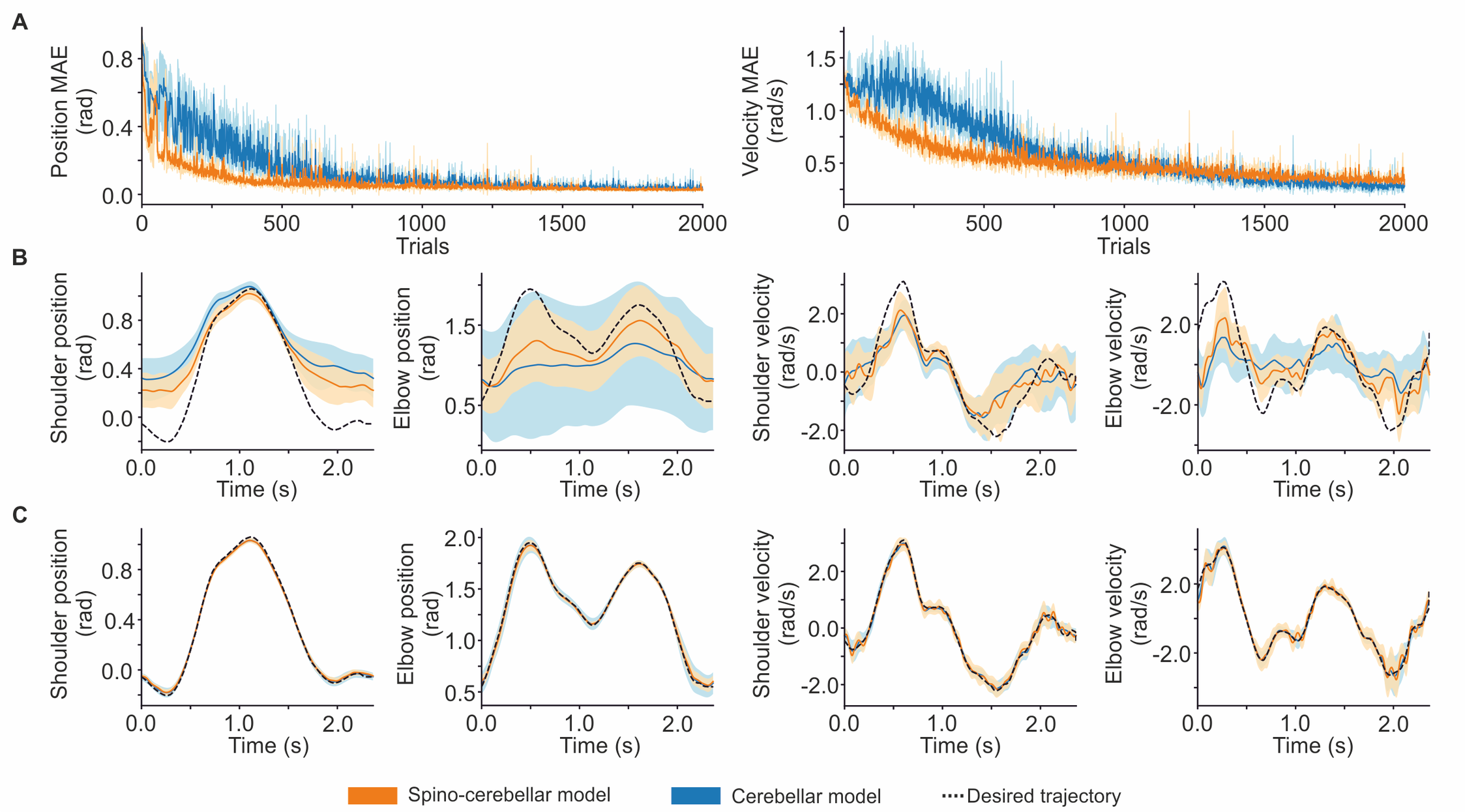

Supplement: S6 Fig — A) Position and velocity mean absolute error (MAE) over the 2000-trial motor adaptation process for both the spino-cerebellar and cerebellar models performing P2’s moderate flexion-extension (2.4 s). B) Joint kinematics of the first 200 trials (mean and standard deviation, std) for both models performing P2’s moderate flexion-extension (2.4 s). C) Joint kinematics of the last 200 trials (mean and std) for both models performing P2’s moderate flexion-extension (2.4 s). (TIF) [file pcbi.1011008.s006.tif]

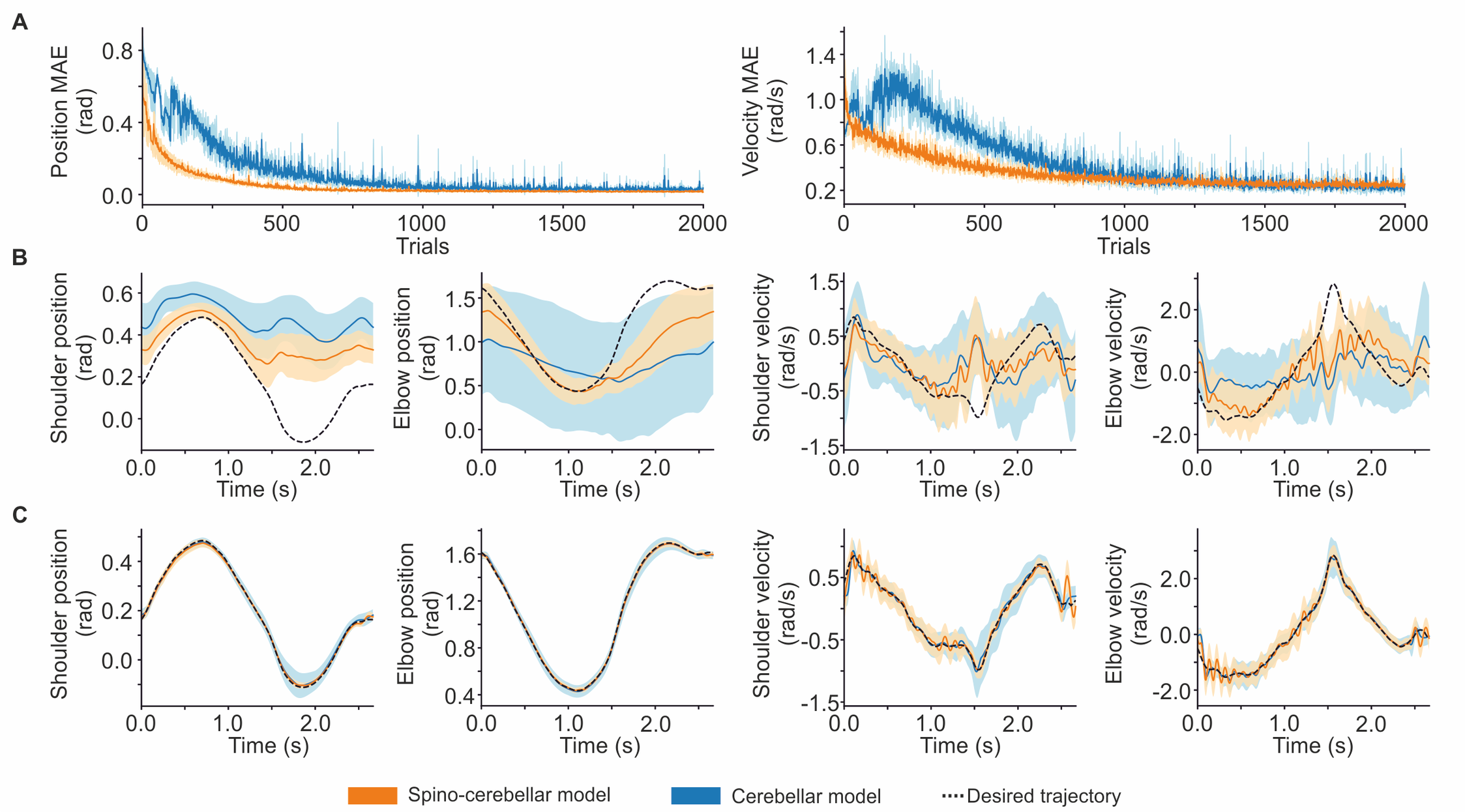

Supplement: S7 Fig — A) Position and velocity mean absolute error (MAE) over the 2000-trial motor adaptation process for both the spino-cerebellar and cerebellar models performing P2’s slow circle trajectory (2.7 s). B) Joint kinematics of the first 200 trials (mean and standard deviation, std) for both models performing P2’s slow circle trajectory (2.7 s). C) Joint kinematics of the last 200 trials (mean and std) for both models performing P2’s slow circle trajectory (2.7 s). (TIF) [file pcbi.1011008.s007.tif]

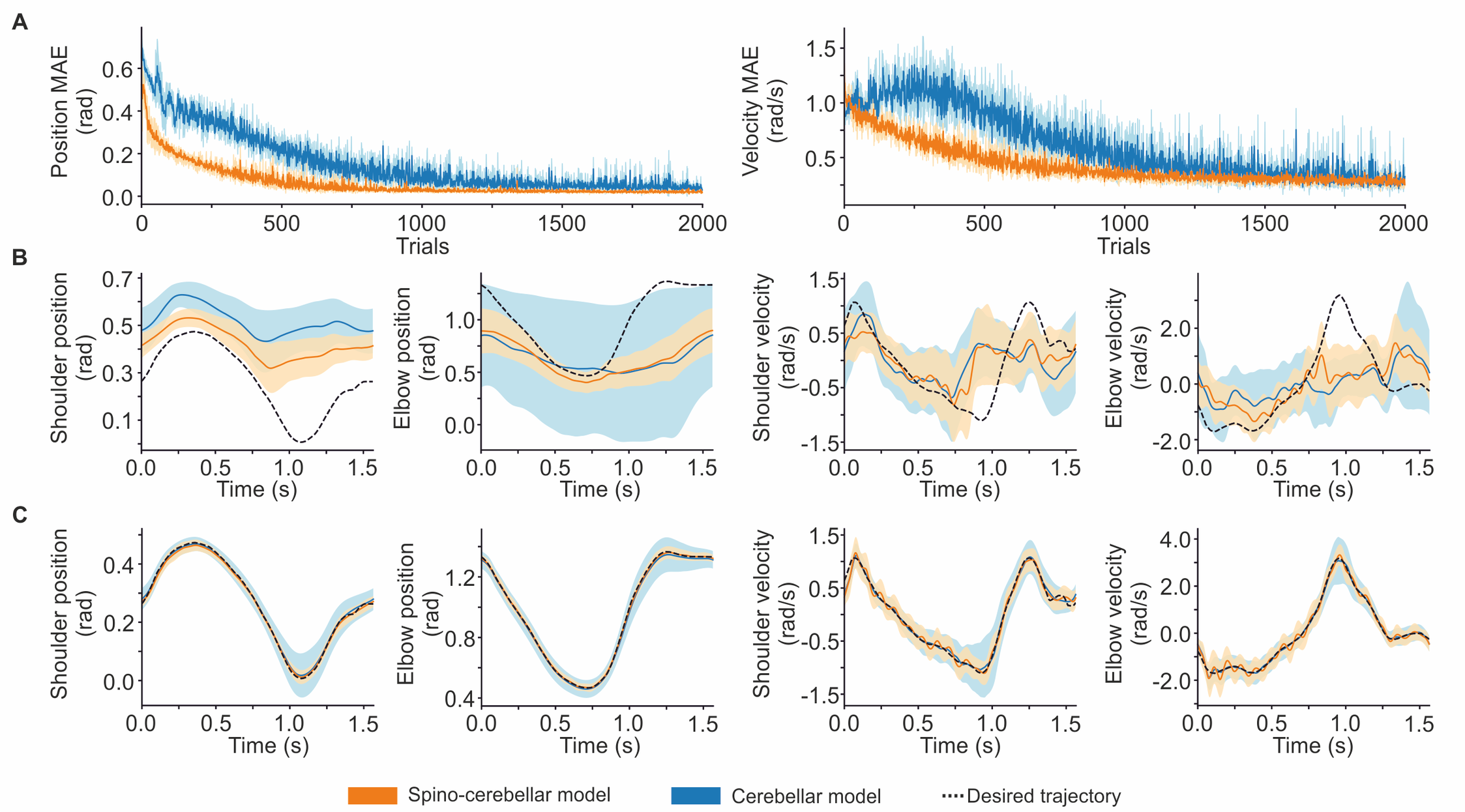

Supplement: S8 Fig — A) Position and velocity mean absolute error (MAE) over the 2000-trial motor adaptation process for both the spino-cerebellar and cerebellar models performing P2’s moderate circle trajectory (1.6 s). B) Joint kinematics of the first 200 trials (mean and standard deviation, std) for both models performing P2’s moderate circle trajectory (1.6 s). C) Joint kinematics of the last 200 trials (mean and std) for both models performing P2’s moderate circle trajectory (1.6 s). (TIF) [file pcbi.1011008.s008.tif]

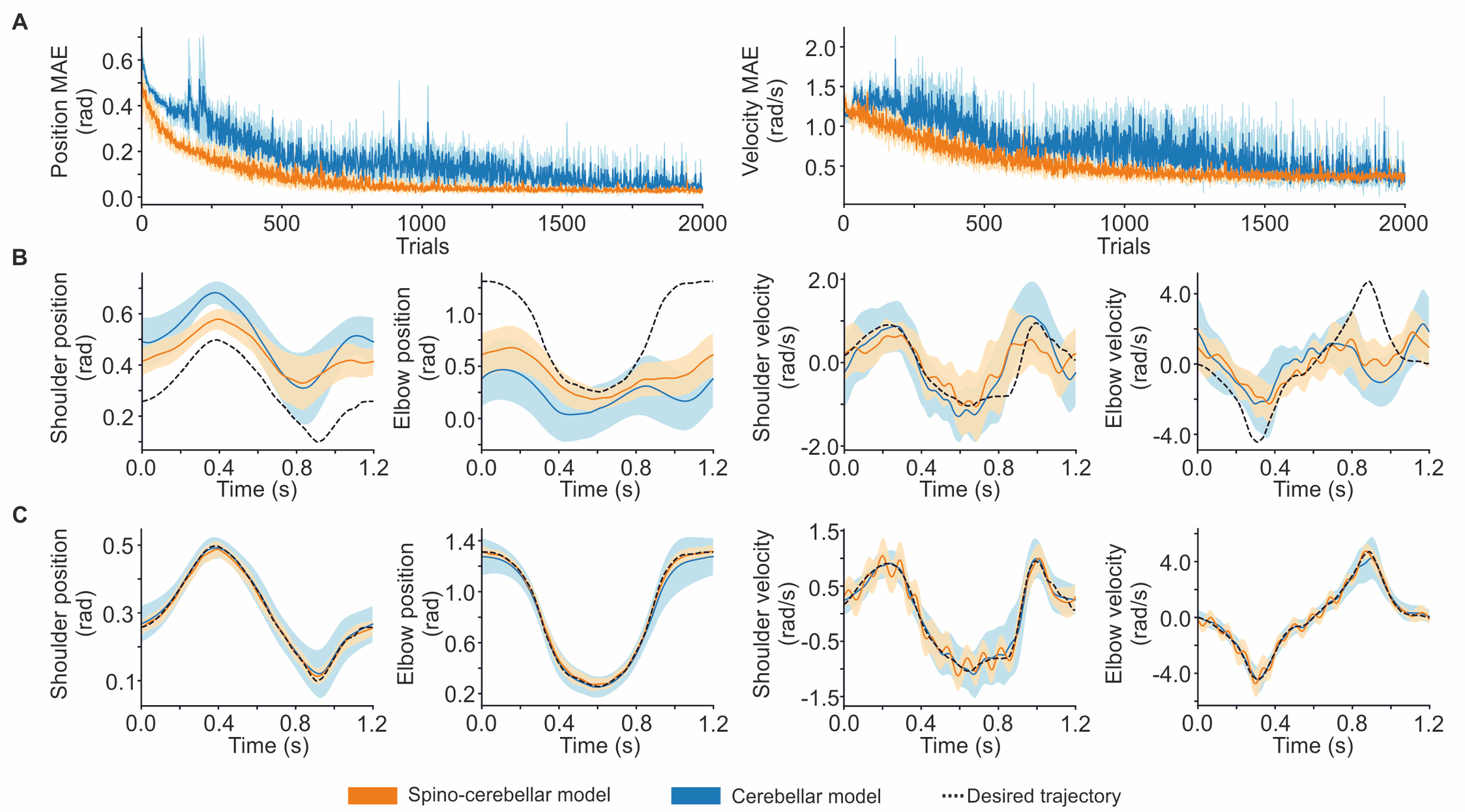

Supplement: S9 Fig — A) Position and velocity mean absolute error (MAE) over the 2000-trial motor adaptation process for both the spino-cerebellar and cerebellar models performing P2’s fast circle trajectory (1.2 s). B) Joint kinematics of the first 200 trials (mean and standard deviation, std) for both models performing P2’s fast circle trajectory (1.2 s). C) Joint kinematics of the last 200 trials (mean and std) for both models performing P2’s fast circle trajectory (1.2 s). (TIF) [file pcbi.1011008.s009.tif]

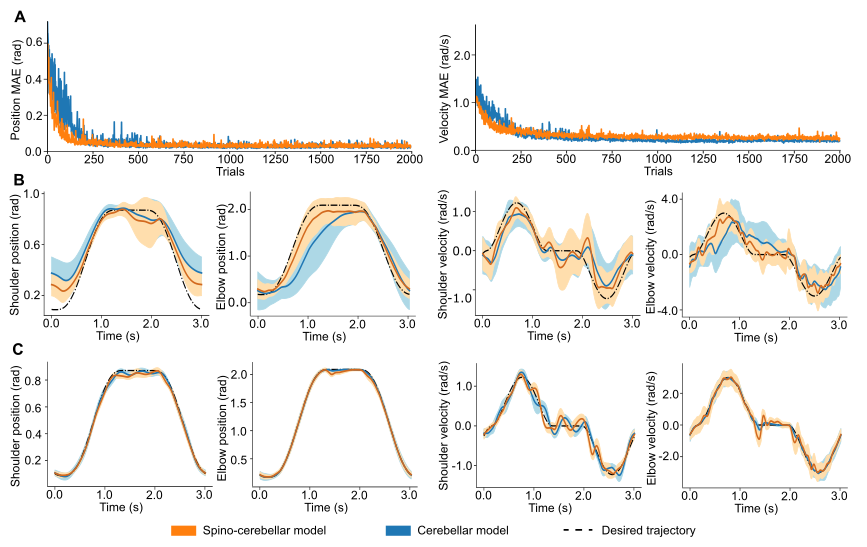

Supplement: S10 Fig — A) Position and velocity mean absolute error (MAE) over the 2000-trial motor adaptation process for both the spino-cerebellar and cerebellar models performing bell-shaped slow flexion-extension (3 s). B) Joint kinematics of the first 200 trials (mean and standard deviation, std) for both models performing bell-shaped slow flexion-extension (3 s). C) Joint kinematics of the last 200 trials (mean and std) for both models performing bell-shaped slow flexion-extension (3 s). (TIF) [file pcbi.1011008.s010.tif]

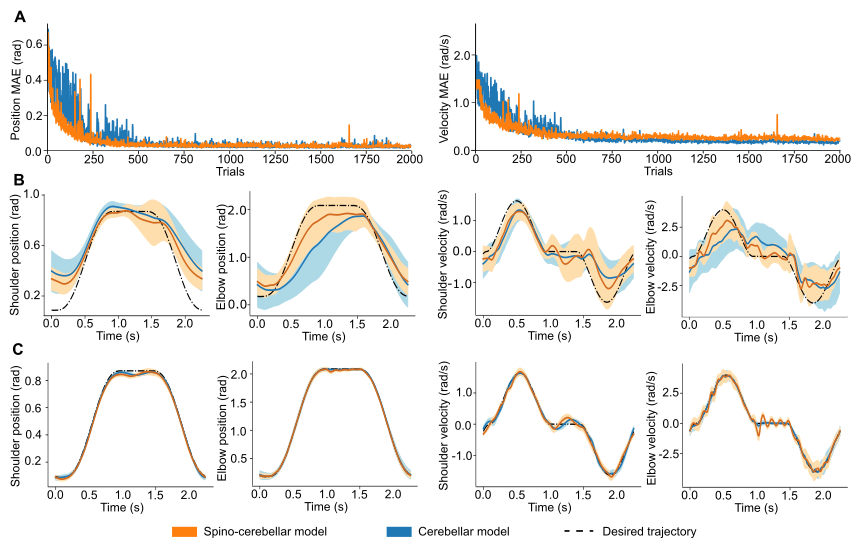

Supplement: S11 Fig — A) Position and velocity mean absolute error (MAE) over the 2000-trial motor adaptation process for both the spino-cerebellar and cerebellar models performing bell-shaped moderate flexion-extension (2.3 s). B) Joint kinematics of the first 200 trials (mean and standard deviation, std) for both models performing bell-shaped moderate flexion-extension (2.3 s). C) Joint kinematics of the last 200 trials (mean and std) for both models performing bell-shaped moderate flexion-extension (2.3 s). (TIF) [file pcbi.1011008.s011.tif]

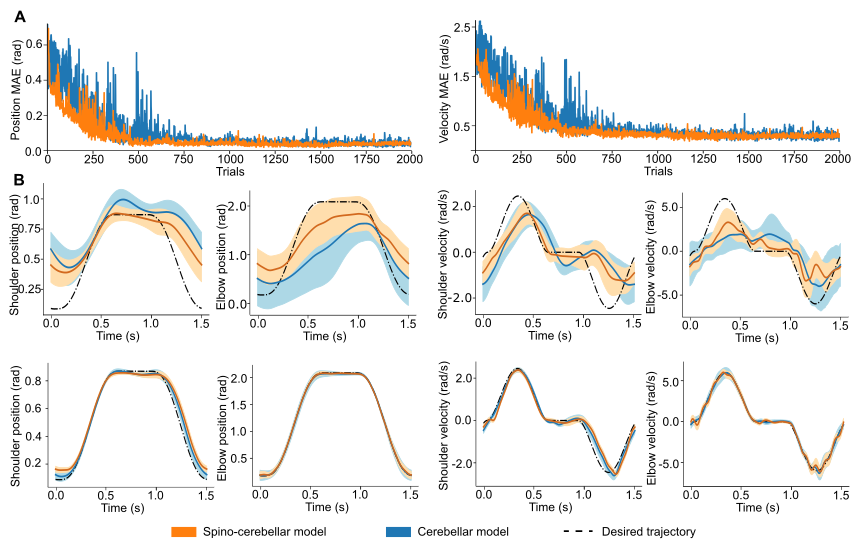

Supplement: S12 Fig — A) Position and velocity mean absolute error (MAE) over the 2000-trial motor adaptation process for both the spino-cerebellar and cerebellar models performing bell-shaped fast flexion-extension (1.5 s). B) Joint kinematics of the first 200 trials (mean and standard deviation, std) for both models performing bell-shaped fast flexion-extension (1.5 s). C) Joint kinematics of the last 200 trials (mean and std) for both models performing bell-shaped fast flexion-extension (1.5 s). (TIF) [file pcbi.1011008.s012.tif]

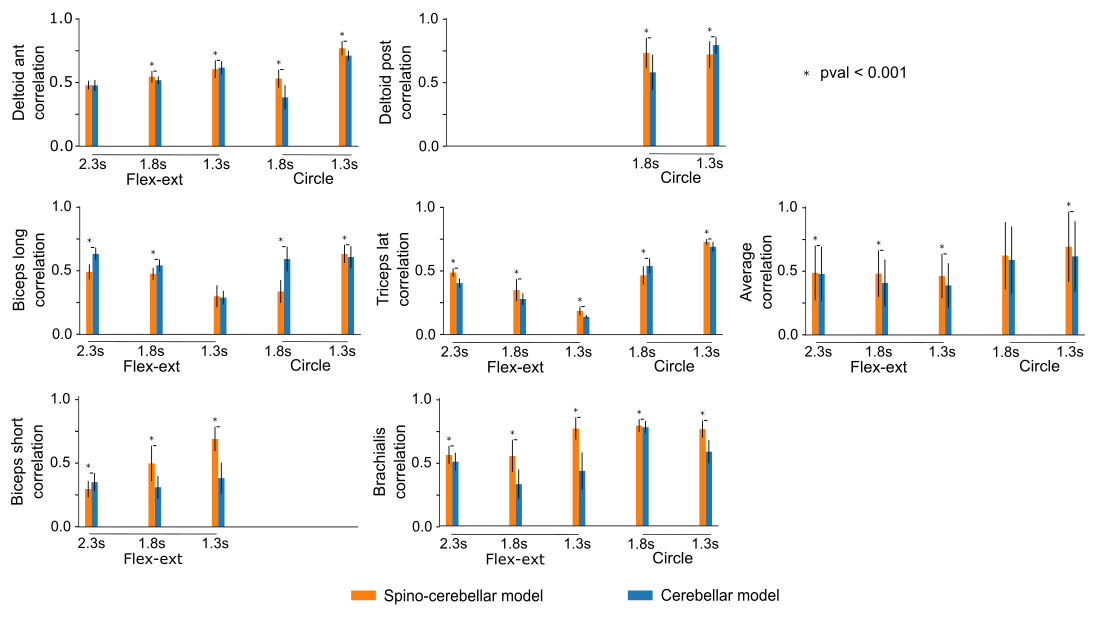

Supplement: S13 Fig — The maximum correlation between activation signals and EMG around lag 0 are displayed for the main activated muscles during each movement type. (TIF) [file pcbi.1011008.s013.tif]

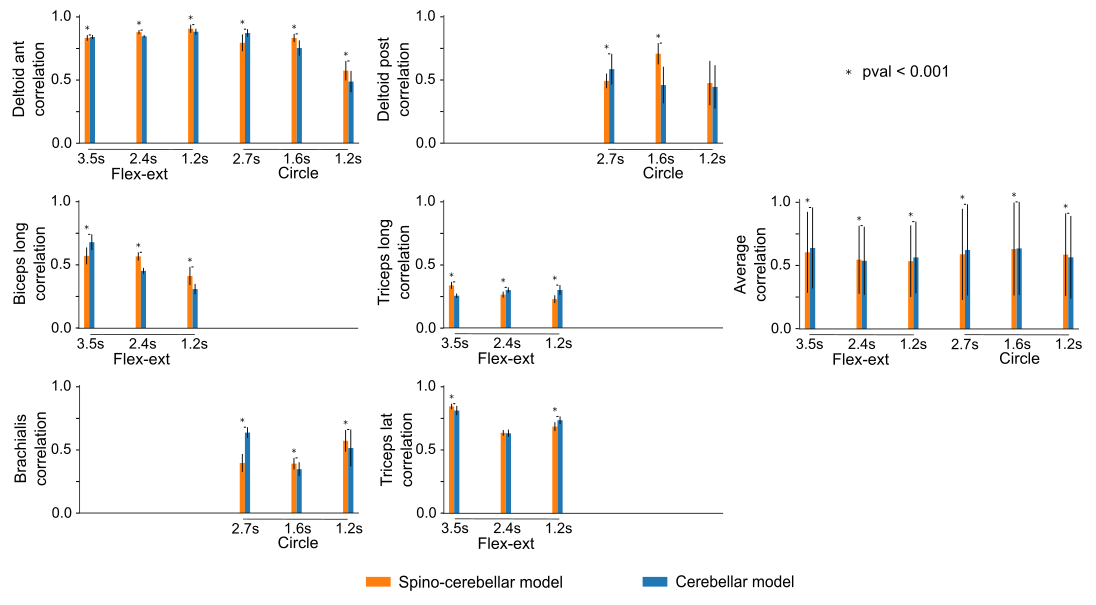

Supplement: S14 Fig — The maximum correlation between activation signals and EMG around lag 0 are displayed for the main activated muscles during each movement type. (TIF) [file pcbi.1011008.s014.tif]

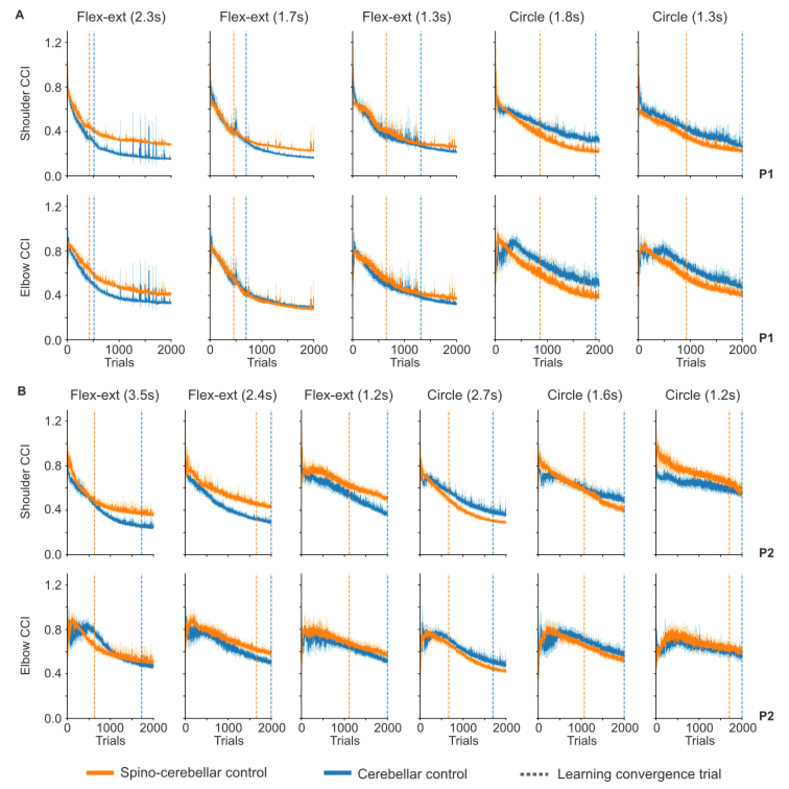

Supplement: S15 Fig — A), B) Joint CCI for both the spino-cerebellar and cerebellar models during the 2000-trial motor adaptation process for all P1 and P2 trajectories, respectively. Top row shows the shoulder CCI, bottom row displays the elbow CCI. (TIF) [file pcbi.1011008.s015.tif]

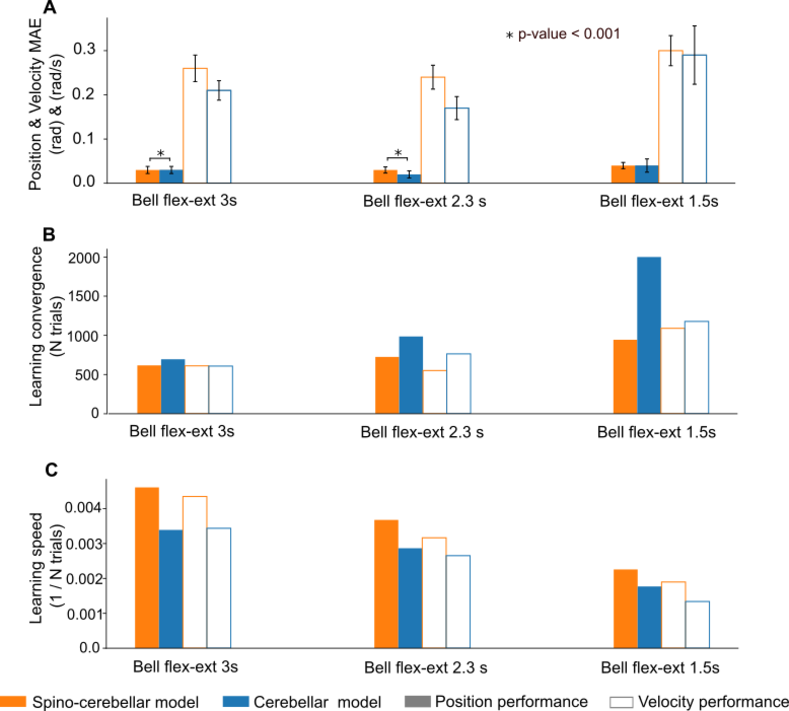

Supplement: S16 Fig — The final performance (MAE), convergence time (from control chart), and learning speed (1 over the number of trials to reach a target MAE value) are compared. (TIF) [file pcbi.1011008.s016.tif]

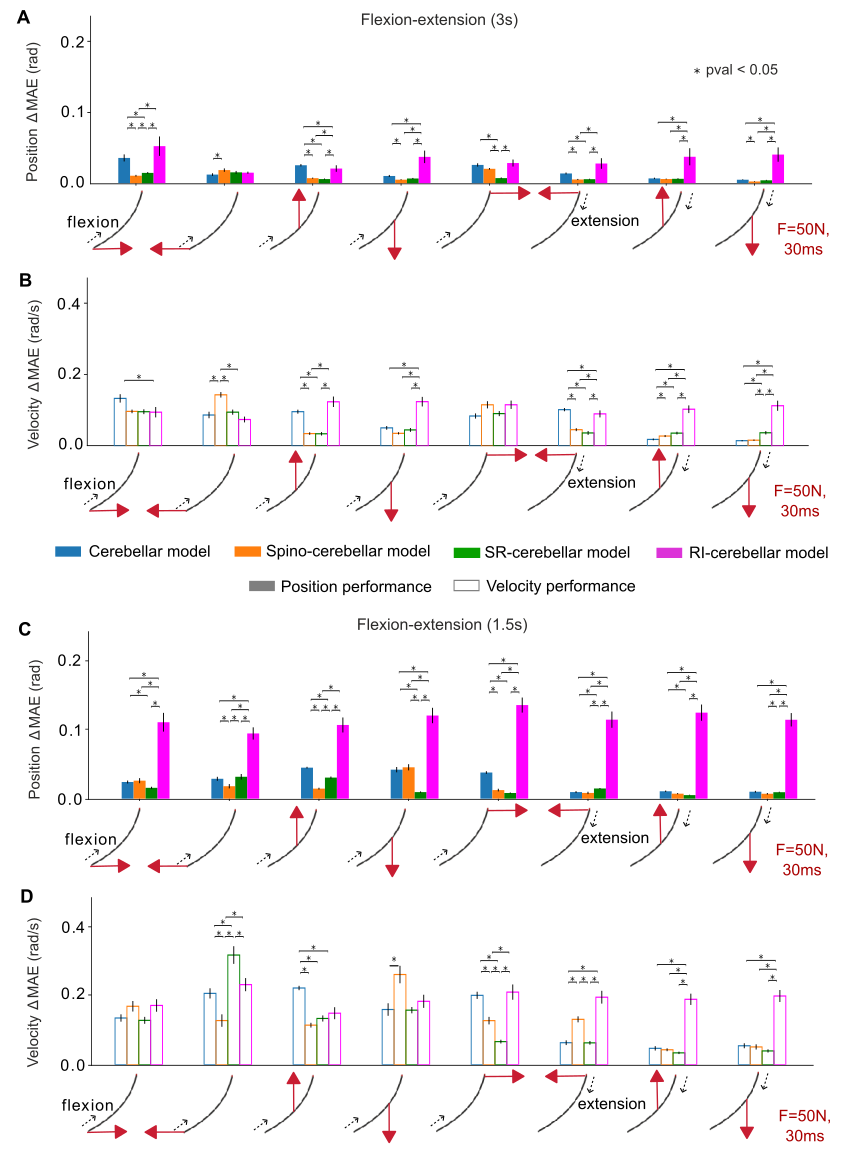

Supplement: S17 Fig — A) Position MAE deviation (ΔMAE¯) caused by all the perturbations applied during the 3s flexion-extension trajectory for the four models. Mean ΔMAE¯ and standard deviation (std) of 50 trials are displayed. B) Velocity MAE deviation (ΔMAE¯) caused by all the perturbations applied during the 3s flexion-extension trajectory for the four models. Mean ΔMAE¯ and std of 50 trials are displayed. C) Position MAE deviation (ΔMAE¯) caused by all the perturbations applied during the 1.5s flexion-extension trajectory for the four models. Mean ΔMAE¯ and std of 50 trials are displayed. D) Velocity MAE deviation (ΔMAE¯) caused by all the perturbations applied during the 1.5s flexion-extension trajectory for the four models. Mean ΔMAE¯ and std of 50 trials are displayed. (TIF) [file pcbi.1011008.s017.tif]
