# Supplementary material for: The spinal cord facilitates cerebellar upper limb motor learning and control; inputs from neuromusculoskeletal simulation
Source: PLoS Comput Biol. 2024 Jan 2;20(1):e1011008. doi: 10.1371/journal.pcbi.1011008 (PMC10786408; doi:10.1371/journal.pcbi.1011008)
Supplement: S1 Text — (DOCX) [file pcbi.1011008.s018.docx]

**S1 Leaky integrate and fire neuron model dynamics**

We used leaky integrate and fire (LIF) neurons to model the cerebellar spiking neural network. Our LIF neurons fired a spike only when their corresponding membrane potential reached the firing threshold, immediately after that, the membrane potential was reset. The LIF neural dynamics was defined by the neuron membrane potential and its excitatory (AMPA and NMDA) and inhibitory (GABA) chemical conductances, as described by:

$$C\cdot\frac{dV}{dt}=I_{int}+I_{ext}$$

 (1)

$$I_{int}=-g_{L}\cdot\left( V-E_{L} \right)$$

 (2)

$$I_{ext}=-\left( g_{AMPA}\left( t \right)+g_{NMDA}\left( t \right)\cdot g_{NMDA_{inf}} \right)\cdot\left( V-E_{AMPA} \right)-g_{GABA}\left( t \right)\cdot\left( V-E_{GABA} \right)$$

 (3)

$$g_{AMPA}\left( t \right)=g_{AMPA}\left( t_{0} \right)\cdot e^{\frac{t-t_{0}}{\tau_{AMPA}}}+\sum_{i=1}^{N} \delta_{AMPA_{i}}\left( t \right)\cdot w_{i}$$

 (4)

$$g_{NMDA}\left( t \right)=g_{NMDA}\left( t_{0} \right)\cdot e^{\frac{t-t_{0}}{\tau_{NMDA}}}+\sum_{i=1}^{N} \delta_{NMDA_{i}}\left( t \right)\cdot w_{i}$$

 (5)

$$g_{GABA}\left( t \right)=g_{GABA}\left( t_{0} \right)\cdot e^{\frac{t-t_{0}}{\tau_{GABA}}}+\sum_{i=1}^{N} \delta_{GABA_{i}}\left( t \right)\cdot w_{i}$$

 (6)

$$g_{NMDA_{inf}}\left( t \right)=\frac{1}{1+e^{62V}\cdot\frac{1.2}{3.57}}$$

 (7)

where

$$C$$

stands for the membrane capacitance;

$$V$$

is the membrane potential;

$$I_{int}$$

 and

$$I_{ext}$$

 are the internal and external currents, respectively;

$$E_{L}$$

 is the resting potential;

$$g_{L}$$

 the conductance responsible for the passive decay term toward the resting potential;

$$g_{AMPA}$$

,

$$g_{NMDA}$$

, and

$$g_{GABA}$$

 are the conductances integrating the contributions received by each receptor type (AMPA, NMDA, or GABA) through individual synapses; being

$$g_{NMDA_{inf}}$$

 the NMDA activation channel. These conductances were defined as decaying exponential functions [114,115], with their values being directly incremented proportionally to the synaptic weight (

$$w_{i}$$

) upon each presynaptic spike arrival (

$$\delta$$

, Dirac delta functions). When the membrane potential reached a threshold

$$V_{thr}$$

, it was then reset to

$$E_{L}$$

 during the refractory period

$$T_{ref}$$

. The configuration parameters of the LIF neurons modelled are summarised in S1 Table.
