# Supplementary material for: The spinal cord facilitates cerebellar upper limb motor learning and control; inputs from neuromusculoskeletal simulation
Source: PLoS Comput Biol. 2024 Jan 2;20(1):e1011008. doi: 10.1371/journal.pcbi.1011008 (PMC10786408; doi:10.1371/journal.pcbi.1011008)
Supplement: Attachment — Submitted filename: response_reviewers.pdf [file pcbi.1011008.s020.pdf]

The authors would like to thank the reviewers for their time spent on reviewing this work and their comments that have helped us improve the manuscript. The comments and suggestions from the reviewers have been taken under consideration and have been addressed in the new version of the manuscript. Below, the authors have done their best to answer the reviewers' questions and comments, point by point. All the changes made in the main text are marked in blue for easy follow up.

Comments

Reviewer #1:

- ***Point 1.1.*** *The authors employ a relatively simple model of spinal reflex pathways and a more elaborated model of cerebellar learning to test whether the spinal circuits enhance cerebellar learning of a range of limb movements compared to direct control of the muscles. All command signals to the spinal cord arise in the model of the cerebellum rather than in a model of motor cortex, which makes the system difficult to interpret in light of conventional distinctions between pyramidal and extrapyramidal control. This point needs better discussion, particularly in the context of the different phenotypes resulting from pathology in those subsystems.*

Answer: We thank the reviewer for helping improve the clarity of our work by addressing the modelling assumptions, which need a proper justification now added to the manuscript. The cerebellum is well-known for its role in motor adaptation and learning, a role mainly supported by the cerebellar ability to acquire internal models of both body-plant dynamics and external objects [*Wolpert, D. M., Miall, R. C., & Kawato, M. (1998). Internal models in the cerebellum. Trends in cognitive sciences, 2(9), 338-347.*]. These internal models are found to be forward or inverse: a forward model maps the sensorimotor state to its predicted motor behaviour; an inverse model maps the desired behaviour to the motor commands that will make it possible. There is evidence supporting the co-existence of both forward and inverse models in the cerebellum [*Manto, M., et al., (2012). Consensus paper: roles of the cerebellum in motor control—the diversity of ideas on cerebellar involvement in movement. The Cerebellum, 11, 457-487.*]. When the cerebellum is modelled as a forward model, it adjusts or modifies the descending motor commands (from the motor cortex) in order to reduce the mismatch between the desired and actual motor behaviour. In such a case, the body-plant dynamics are learnt at higher brain areas and the cerebellum operates as a corrective mechanism in addition to those already learnt dynamics. When an inverse model, the cerebellum does not depend on descending motor commands, but rather can directly provide the whole motor output. As put by Ito in [*Ito, M. (1997). Cerebellar microcomplexes. International review of neurobiology, 41, 475-487.*]: "With an inverse dynamics model in the cerebellum, however, one would be able to perform a movement automatically without conscious thought because command signals would bypass the motor cortex".

In our work, the cerebellum is implemented as an inverse model, therefore putting it in a worst-case scenario in which it has to learn the entire body-plant dynamics. The purpose of this approach is that it allows directly addressing the influence that the spinal cord (SC) has on cerebellar motor learning.

If a forward model was used instead, a better tracking performance would be probably obtained, but at the cost of hampering the evaluation of the SC influence on cerebellar performance. The SC, due to its direct and fast action on muscle activation, constitutes in itself a significant component of the body dynamics and would therefore influence the motor cortex output. If the SC control mechanisms were already being accounted for at higher brain areas, it would then be difficult to evaluate and weigh the

SC effect on cerebellar learning. Within our framework, we can include or remove the SC from the control loop to compare the spino-cerebellar and cerebellar cases and directly address what effect the SC has on cerebellar motor learning.

A new subsection called "***Cerebellum and spinal cord modelling assumptions***" has been added to the manuscript *Discussion* section, lines 477-560. The full new subsection has been added to the end of this review letter for simplicity, please refer to paragraphs 2 and 3 which address the modelling assumptions discussed above.

We thank the reviewer for the feedback and hope to have addressed the raised concerns.

- ***Point 1.2.*** *The cerebellar model is organized by individual joints and the muscles that act upon them, rather than whole limb kinematics of the limb as proposed by Poppele and Bosco (2001; Physiological reviews, 2:539-568) from electrophysiological studies of the spinocerebellar tracts. The convergent inputs in the cerebellar parallel fibers and divergent outputs of the cerebellar deep nuclei seem incompatible with the joint-based architecture of this cerebellar model. Presumably this shows up in the clustering of synaptic weight distributions in Fig. 5A-B but this isn't explained. Whether this unphysiological simplification affects the validity of the conclusions needs further discussion.*

Answer: We appreciate this feedback which we believe will further support the findings of our work by clarifying the modelling assumptions. The referenced work by Poppele and Bosco (2001) states that within the dorsal spinocerebellar tract (DSCT) there is a subpopulation of neurons representing whole limb parameters and another equally-sized subpopulation of neurons representing joint-related signals. The authors posit that this halved structure "might underlie some kind of comparison between end point-related and joint-related signals", thus contributing to an early processing stage of sensory information in the spinal cord circuitry, which ultimately facilitates a reference frame for whole limb kinematics. In our study, the focus was not on this early processing of information in the DSCT, which is only one of the many inputs to the cerebellum as stated by the referenced authors. Rather, we focused on how the implemented spinal cord mechanisms (i.e., stretch reflex and reciprocal inhibition) impact cerebellar motor adaptation. Nonetheless, the cerebellum did account for whole limb kinematics as the driving force behind the motor adaptation process. The structure of our cerebellar model involves assigning a cerebellar microcomplex to each joint, following Ito's microcomplex theory [*Ito, M. (1997). Cerebellar microcomplexes. International review of neurobiology, 41, 475-487.*]. Whilst the input sensory signals and output motor signals are joint-based, i.e., divided in a modular organisation of different cerebellar functional units [*Apps, R., et al., (2018). Cerebellar modules and their role as operational cerebellar processing units. The Cerebellum, 17, 654-682.*], the cerebellar learning at the granule cells - Purkinje cells synapses (GC-PC), in contrast, considers the entire limb kinematics.

GCs are believed to perform a recoding of sensory inputs [*Carrillo, R. R., et al., (2018). A metric for evaluating neural input representation in supervised learning networks. Frontiers in Neuroscience, 12, 913.; Cayco-Gajic, N. A., et al., (2017). Sparse synaptic connectivity is required for decorrelation and pattern separation in feedforward networks. Nature communications, 8(1), 1116.*], ensuring an univocal and unambiguous representation of the kinematic state of the limb. GCs, in turn, transmit this sensory state to PCs which, driven by the climbing fibres (CFs) instructive action, adapt their activity to cope with the desired motor behaviour. Importantly, PCs are innervated by a massive number of GCs (~175000

GC-PC synapses are estimated in the rat cerebellar cortex for each PC [*Napper, R. M. A., & Harvey, R. J. (1988). Number of parallel fiber synapses on an individual Purkinje cell in the cerebellum of the rat. Journal of Comparative Neurology, 274(2), 168-177.*]). Amongst that massive GC-PC innervation, PCs receive excitatory inputs from GCs within the very same microcomplex and also from GCs belonging to other microcomplexes, thus allowing the linkage of different cerebellar microcomplexes through GC-PC synapses [*Valera, A. M., et al., (2016). Stereotyped spatial patterns of functional synaptic connectivity in the cerebellar cortex. Elife, 5, e09862.; Apps, R., et al., (2018). Cerebellar modules and their role as operational cerebellar processing units. The Cerebellum, 17, 654-682.*]. Within that massive GC-PC connectivity, statistically identical PCs (i.e., receiving statistically identical inputs) have been spotted as a means to smooth the cerebellar output, thus generating a more reliable output able to cope with cerebellar signal-to-noise challenges [*Apps, R., et al., (2018). Cerebellar modules and their role as operational cerebellar processing units. The Cerebellum, 17, 654-682.*].

In our model, the aforementioned factors, along with the computational limitations, were represented by each PC receiving excitatory input from all GCs, not just from those associated with the very same microcomplex. Thus each PC receives the entire sensory information from GCs, and a specific teaching/instructive signal through a particular CF corresponding to the same microcomplex, in accordance to CF-PC one-to-one synapses [*Eccles, J. C., et al., (1966). The excitatory synaptic action of climbing fibres on the Purkinje cells of the cerebellum. The Journal of physiology, 182(2), 268-296.*]; i.e., synaptic plasticity at PCs is affected by the sensory information from all joints, enabling the cerebellum to acquire a comprehensive internal model representation of the upper-limb, rather than a joint-specific representation. Therefore, despite the microcomplex structure being based on individual joints, the ensemble activity of GCs across PCs allows for motor adaptation to account for the entire limb kinematics.

As suggested, to provide the reader with a further description of this and other modelling choices, and to address the aforementioned, a new subsection called "***Cerebellum and spinal cord modelling assumptions***" has been added to the manuscript *Discussion* section, lines 477-560. The full new subsection has been added to the end of this review letter for simplicity, please refer to paragraphs 5 and 6 which address the modelling assumptions discussed above.

We appreciate the feedback and hope this clarifies the model implementation

- ***Point 1.3.*** *The modeled cerebellar output controls the motoneurons directly rather than modulating the gains of the homonymous and antagonist reflexes, which would be more consistent with the known connectivity and function, as now intimated in the Discussion. It is not clear how the arbitrarily chosen synaptic weights correspond to reflex gains. None of the well-known heteronymous excitatory stretch projections are modeled. Whether this affects the validity of the conclusions needs further discussion.*

Answer: We thank the reviewer for this comment, which will help to further clarify our modelling assumptions. Some modelling aspects were simplified to facilitate the interpretation of the results. This is particularly true for the case of the cerebellar output commands directly driving the motoneuron activations. In our work, we distinguished between two control cases: i) the spino-cerebellar integration; ii) the cerebellum operating in isolation. This spino-cerebellar vs. cerebellar control framework provided a clear context that allowed us to study the influence of the spinal cord (SC) on cerebellar motor learning. Whilst case (i) implies that cerebellar motor learning is

inevitably affected by the SC, case (ii) provides a reference baseline, with the cerebellar model being the sole nervous region responsible for motor behaviour. Therefore, in case (ii), the cerebellar output commands are required to directly control muscle activation. To facilitate a direct comparison between scenarios (i) and (ii), in case (i) we maintained the cerebellar descending commands to the SC as direct control signals acting upon motoneurons. Thus, the descending cerebellar commands were equivalent in both cases (i) and (ii), allowing us to directly study the influence of the implemented SC mechanisms (i.e., stretch reflex and reciprocal inhibition) on cerebellar motor learning.

Regarding the synaptic weights of the spinal pathways, we set them according to the ratio between the amplitudes of excitatory postsynaptic potentials (EPSPs) from Group la afferents reported in [*Fleshman, James W., et al., "Homonymous projection of individual group Ia-fibers to physiologically characterised medial gastrocnemius motoneurons in the cat." journal of Neurophysiology 46.6 (1981): 1339-1348*] and inhibitory postsynaptic potentials (IPSPs) from Group la inhibitory interneurons observed in [*Stuart, G. J., and S. J. Redman. "Voltage dependence of Ia reciprocal inhibitory currents in cat spinal motoneurons." The Journal of physiology 420.1 (1990): 111-125*]. Thus, the synaptic weights of our spinal model are chosen to reproduce physiological connectivity.

Regarding the missing heteronymous projections highlighted by the reviewer, we opted for simplifying the spinal network for the sake of interpretability. The presence of heteronymous projections in the human upper limb has been effectively reported in [*McClelland, V. M., S. Miller, and J. A. Eyre. "Short latency heteronymous excitatory and inhibitory reflexes between antagonist and heteronymous muscles of the human shoulder and upper limb." Brain research 899.1-2 (2001): 82-93*]. According to this study, inhibition occurs significantly more frequently than excitation between antagonist muscle pairs, with a higher frequency of inhibition received from antagonists muscles compared to muscles acting across a different joint. These findings are aligned with our SC model, as we incorporated reciprocal inhibition pathways to capture this pattern.

In *McClelland et al.* study, it was also observed bi-directional excitation; however in [*Pierrot-Deseilligny, E., & Burke, D. (2012). The circuitry of the human spinal cord: spinal and corticospinal mechanisms of movement. Cambridge University Press.; Cavallari, P., & Katz, R. (1989). Pattern of projections of group I afferents from forearm muscles to motoneurons supplying biceps and triceps muscles in man. Experimental Brain Research, 78, 465-478*] it was noted that proximal to distal heteronymous Ia connections (from the arm to forearm) were absent, whilst wide connections from distal to proximal muscles were present, although weaker from proximal to shoulder muscles. [*Pierrot-Deseilligny, 2012*] suggested that these stronger connections at wrist and elbow level may assist the hand muscles in grasping and lifting movements by providing stability to the corresponding joints. Consequently, heteronymous excitatory projections are reported to have less significance than heteronymous inhibitory projections in human upper limb movements. However, we acknowledge that heteronymous excitatory projections are an essential aspect of the overall motor control system, and their exclusion in our SC model does not diminish their significance. It is important to note that incorporating the heteronymous excitatory projections to our SC model would have added complexity, making the results more challenging to interpret and analyse. We opted for a simplified approach that allowed us to examine the inhibitory pathways more effectively. We believe that, in future modelling work, further physiological investigation would be interesting to gain understanding of the specific role and dynamics of these excitatory projections and their interactions with inhibitory pathways in upper limb movements.

To provide the reader with a deeper understanding of the modelling choices and to address the aforementioned, a new subsection called "***Cerebellum and spinal cord modelling assumptions***" has

been added to the manuscript *Discussion* section, lines 477-560. The full new subsection has been added to the end of this review letter for simplicity. Please refer to paragraph 4 which addresses cerebellar direct control of motoneurons, and paragraphs 7 and 8 which address the spinal cord modelling assumptions discussed above.

- ***Point 1.4.*** *The authors discuss the importance of cocontraction but this is difficult to appreciate from the highly extracted cocontraction indices by joint, especially in view of the biarticular muscles that are known to have special functions related to energy-efficient transfer of momentum (van Ingen Schenau et al., 1994, Differential use and control of mono-and biarticular muscles. Human Movement Science, 13:495-517).*

Answer: The joint cocontraction index (CCI) was obtained using the method developed in [*Rudolph, K. S., et al., (2000). Dynamic stability after ACL injury: who can hop?. Knee Surgery, Sports Traumatology, Arthroscopy, 8(5), 262.*]. This method associates high CCI values to high levels of activation of both agonist and antagonist muscle groups during the performance of the motor task. Conversely, low CCI values indicate poor activation of both agonist and antagonist muscles, or high activation of one muscle group and low activation of the opposing group. Importantly, this method for extracting CCI was later assessed in [*Li, G., et al., (2021). How well do commonly used co-contraction indices approximate lower limb joint stiffness trends during gait for individuals post-stroke?. Frontiers in Bioengineering and Biotechnology, 8, 588908.*] proving a good correlation between CCI and joint stiffness.

During the early stages of learning, it has been reported that joint stiffness is higher to facilitate motor adaptation [*Franklin, D. W., et al., (2003). Adaptation to stable and unstable dynamics achieved by combined impedance control and inverse dynamics model. Journal of neurophysiology, 90(5), 3270-3282.*]. Thus, the joint CCI used in our study (which correlates well with joint stiffness) allowed us to contextualise the results within the framework of spinal cord facilitation of cerebellar motor adaptation. This facilitation occurs at both kinematic and synaptic levels, which are the key findings of our work. For a more detailed discussion on the relationship between the reported CCI values and the development of internal models in the cerebellum, please refer to *Point 1.6* of this review letter.

Regarding biarticular muscles, in our study we employed two biarticular muscles, namely the biceps long and the triceps long, which were fully accounted for when computing the CCI for both the shoulder and elbow. These biarticular muscles may indeed serve special functions related to efficient transfer of power between joints as pointed out by the reviewer [*van Ingen Schenau et al., 1994, Differential use and control of mono-and biarticular muscles. Human Movement Science, 13:495-517*], whilst contributing to movement stabilisation through cocontraction, as shown in [*Gribble, Paul L., et al. "Role of cocontraction in arm movement accuracy." Journal of neurophysiology 89.5 (2003)*]. Remarkably, Gribble et al. showed high cocontraction in the shoulder, elbow and biarticular pairs during accurate arm movements whilst Kawai et al. [*Y. Kawai, R. J. Downey, H. Kawai and W. E. Dixon, "Co-contraction of antagonist bi-articular muscles for tracking control of human limb," 2014 American Control Conference, Portland*] modelled the cocontraction of biarticular muscle pairs and highlighted its role in stabilising the joint, and controlling the direction of the output force.

We thank the reviewer for the comment and we have now made efforts to clarify the CCI extraction method and address the importance of biarticular muscles. The following modifications have been made to the manuscript.

Manuscript lines 279-281, *Results section 2.4 Spino-cerebellar and cerebellar outcome in muscle space*:

"*To overcome this, we further studied performance in muscle space using the joint cocontraction index (CCI), which unifies muscle activity per joint and provides a more comprehensive analysis (refer to the Methods section for details on the CCI computation procedure).*"

Manuscript lines 857-865, *Methods* section *4.6.5 Measuring muscle space performance*:

"*It is worth noting that biarticular muscles play specialised roles in energy-efficient transfer of momentum between joints [van Ingen Schenau et al., (1994)], whilst also contributing to movement stabilisation through cocontraction [Gribble, Paul L., et al. (2003)]. Biarticular muscles were fully accounted for when computing the CCI for both the shoulder and elbow. The joint CCI was computed using the method developed in [Rudolph, K. S., et al., (2000).], by which high CCI values correspond to high levels of activation of both agonist and antagonist muscle groups, and low CCI values indicate poor activation of both muscle groups, or high activation of one muscle group and low activation of the opposing group. Importantly, this method for extracting CCI was later evaluated in [Li, G., et al., (2021)], demonstrating a strong correlation between CCI and joint stiffness.*"

- ***Point 1.5.*** *EMG data that are buried in the Supplemental Figures are confusing and concerning. For circular movements in S13B, subject P1 shows highly reciprocal EMG in anterior vs. posterior deltoid but very high cocontraction index for shoulder in D. For the same movements by P2 in Fig. S14, the EMGs show abnormally high cocontraction of anterior and posterior deltoids but lower shoulder CCI at the same point of 60-70% of movement cycle. The correlations between model muscle activations and recorded EMG from both P1 and P2 are modest for both models but said to all be statistically different between models. Given that the models were all trained on kinematics rather than EMG and the subjects have such different EMG strategies, that doesn't seem possible.*

Answer: We would like to express our gratitude to the reviewer for bringing to our attention the typo of inadvertently inverting panels A and B of S13 and S14. Furthermore, we have made revisions and merged the two figures into a new integrative figure displaying all the recorded EMG signals and their corresponding CCI. The figure has been included as part of the main text, new Figure 5. The following changes have been made to the manuscript:

Manuscript lines 263-269, *Results* section *2.4 Spino-cerebellar and cerebellar outcome in muscle space*:

"*We compared the recorded EMG envelopes to the main activated muscles from the spino-cerebellar and cerebellar models during performance of P1 and P2 trajectories. Fig 5 displays all the participants' recorded EMG signals and corresponding joint cocontraction index (CCI). Fig 6A illustrates the deltoid posterior (DELTpost) and brachialis (BRA) muscles during P1 slow circle performed by both the spino-cerebellar and cerebellar models, whilst deltoid anterior (DELTant) and triceps lateral (TRIlat) muscles are depicted for P2 fast flexion-extension.*"

Manuscript page 14, *new Figure 5 and caption*:

"***EMG recordings obtained from both P1 and P2 during flexion-extension and circular movements. A)** EMG recordings obtained from P1 during flexion-extension movements and **B)** circular movements. The EMG signals of all recorded movements performed at different speeds are interpolated and displayed together to highlight the main activation patterns. Muscles are grouped together by joint (left column for shoulder, right column for elbow) and direction of motion (top row for flexor muscles, bottom row for extensor muscles). For simplicity, biarticular muscles (i.e., biceps long and triceps long) are only displayed on the shoulder group (left column). **C)**, **D)** Resulting joint cocontraction index (CCI) from P1 during flexion-extension and circular movements, respectively. **E)** EMG recordings obtained from P2 during flexion-extension movements and **F)** circular movements. **G)**, **H)** Resulting joint CCI from P2 during flexion-extension and circular movements, respectively.*"

The differences between P1 and P2 EMG strategies are not solely attributed to interindividual variations in muscle recruitment patterns during multi-joint movements [*Hilt, P. M., et al., (2020). Motor recruitment during action observation: effect of interindividual differences in action strategy. Cerebral Cortex, 30(7), 3910-3920.*]. These differences also arise from the fact that both subjects did not execute identical movements in terms of kinematics (for specific joint kinematics of each P1&P2 performed movement, please refer to Fig 2 and S1-S9). This was briefly discussed in the *Methods* section - *4.6.5 Measuring muscle space performance* and has now been further extended as follows:

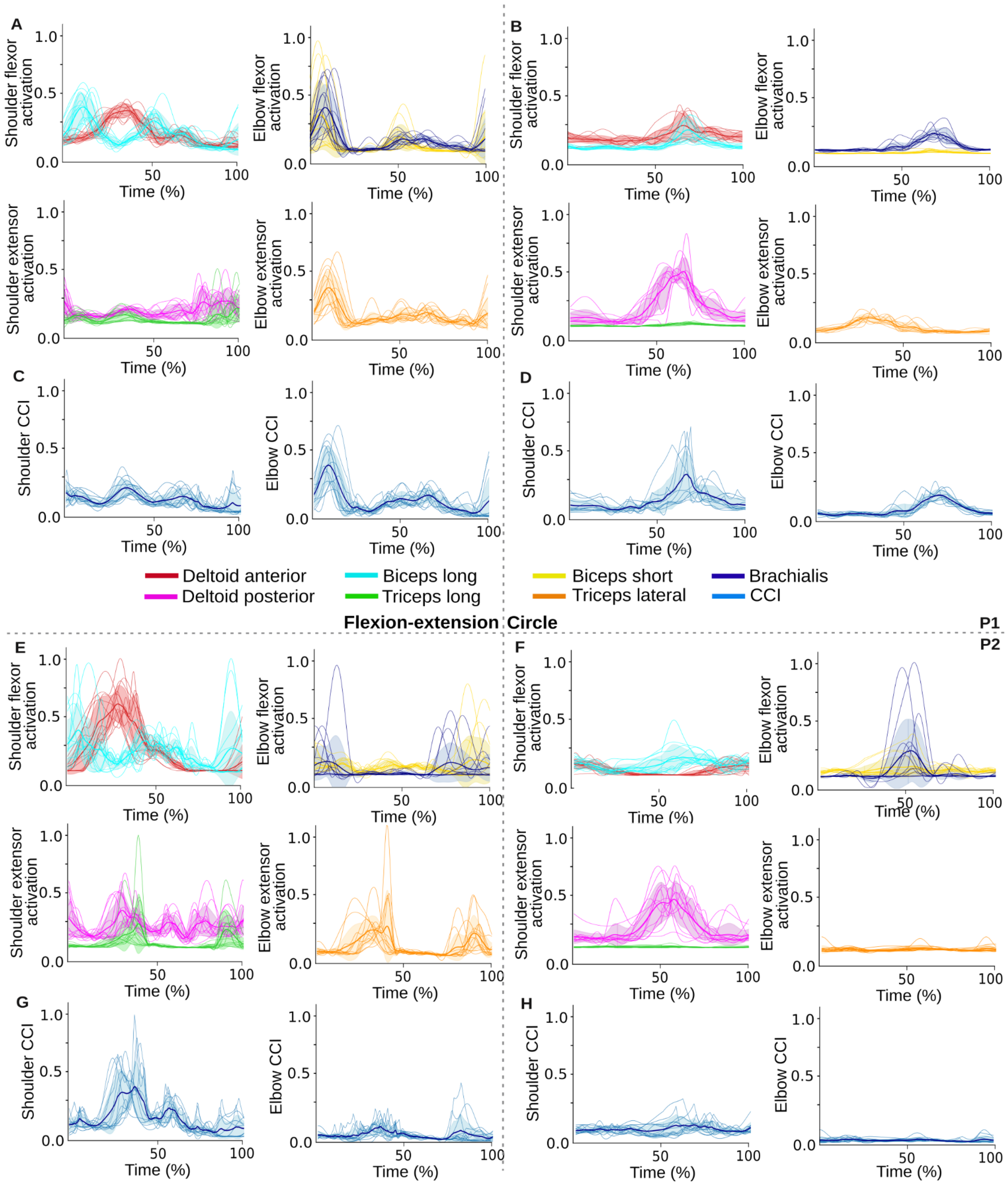

Manuscript lines 837-846, *Methods* section *4.6.5*:

"*Thus, there is inter-participant variability in muscle patterns, as previously described for multi-joint movements in [Hilt, P. M., et al., (2020)] (the participants' recorded EMG data and the corresponding main muscle patterns are displayed in Fig 5). Additionally, the differences in EMG strategies between participants were influenced by variations in movement kinematics, i.e., the participants did not perform the exact same movements (refer to Fig. 2 and S1-S9 for the joint kinematics of each P1 and P2 movement). It is worth noting that during flexion-extension movements, P2 exhibited smaller shoulder extension and larger elbow flexion compared to P1, resulting in greater activation of the BICshort and BRA muscles, and lesser activation of the DELTpost and TRIlong*

muscles. Similarly, during circular trajectories, P2 exhibited greater elbow flexion corresponding to larger activation of BICshort and BRA muscles."

Regarding the simulation results, our models were trained exclusively using kinematics to highlight the influence of the SC in modulating muscle activity. If the cerebellar network had been trained using EMG information, both the spino-cerebellar and cerebellar models would exhibit similar muscle recruitment strategies learnt by the cerebellum. In other words, the influence of the SC in muscle activity modulation would be diminished. By not incorporating EMG information in cerebellar learning, any disparity found in the muscle recruitment strategies of the spino-cerebellar vs. cerebellar models can be attributed to the presence or absence of the SC. The correlation between muscle activation and recorded EMG data of each of the two models (spino-cerebellar vs. cerebellar) demonstrated that the SC model yielded a more physiologically similar muscle recruitment strategy. This analysis helped us validate the SC facilitation of cerebellar motor learning, discussed in the first sections of the manuscript. We have further elaborated on this aspect in the manuscript as follows:

Manuscript lines 740-744, *Methods* section *4.5 Cerebellar instructive signal*:

"*Both the spino-cerebellar and cerebellar models were trained exclusively using kinematics to highlight the influence of the SC in modulating muscle activity. If EMG data had been incorporated in training the models, the cerebellar network would adapt and learn to replicate the recorded muscle patterns, resulting in similar muscle recruitment strategies for both the spino-cerebellar and cerebellar models. In other words, the influence of the SC in muscle activity modulation would be diminished.*"

- ***Point 1.6.*** *The spinal circuitry model employed here is a tiny subset of the spinal circuitry that corresponds to aspects of servocontrol. Raphael et al. (2010; J. Neurosci. 30:9431-9444) showed that incorporation of even more of the known proprioceptive and fusimotor circuitry in a simpler model of a 2 DoF wrist improved the reinforcement learning of a range of tasks while reducing unphysiological cocontraction observed with servocontrol reflexes, the opposite of what was found here.*

Answer: We appreciate the reviewer's feedback, and we have made efforts to improve the clarity of the cocontraction results. There is biological evidence supporting higher cocontraction levels during the early stages of learning, e.g., infants exhibit higher cocontraction during stepping motions compared to adults, and it is subsequently reduced with practice [*Yang, J. F., et al., (1998). Infant stepping: a method to study the sensory control of human walking. The Journal of physiology, 507(3), 927-937.*]; in the case of the upper limb, higher cocontraction (leading to higher joint stiffness) has been reported during learning and adaptation to new dynamics [*Franklin, D. W., et al., (2003). Adaptation to stable and unstable dynamics achieved by combined impedance control and inverse dynamics model. Journal of neurophysiology, 90(5), 3270-3282; Osu, R., et al., (2002). Short-and long-term changes in joint co-contraction associated with motor learning as revealed from surface EMG. Journal of neurophysiology, 88(2), 991-1004.*]. These findings suggest that higher cocontraction during early learning stages enhances the learning rate and facilitates the acquisition of internal models, enabling a subsequent gradual reduction of the cocontraction levels [*Heald, J. B., et al., (2018). Increasing muscle co-contraction speeds up internal model acquisition during dynamic motor learning. Scientific reports, 8(1), 16355.*]. In our work, both the spino-cerebellar and the cerebellar model exhibit a gradual decrease in joint cocontraction indexes (CCI) as the motor adaptation process evolves. As a result, both models

present a biologically plausible behaviour in the broader context of reducing cocontraction through learning. To illustrate this point a new supplementary figure has now been included depicting the evolution of CCI throughout the motor adaptation process (see below). Please note that due to computational constraints each simulation was limited to 2000 trials, and we used convergence of the kinematic performance as a learning metric. However, as depicted in the new figure, the tendency of the cocontraction continues to decrease at the end of the 2000 trials. Notably, at that point, the spino-cerebellar model exhibits higher CCI values than the cerebellar model in some cases and lower CCI values in others. Consequently, we could not draw definitive conclusions on the final CCI values. Instead, our focus was on the CCI values as the kinematic adaptation progressed, thus establishing a connection between CCI values and our previously discussed results regarding the SC facilitating cerebellar motor learning. We apologise if this was not clearly stated in the previous version of the article.

The CCI values presented in the main text correspond to the first 200 trials at which a stable kinematic performance was achieved. This point indicates the convergence of the motor adaptation process (see Methods) and represents the stage at which an accurate internal model was developed.

This information was initially stated in the figure caption, however, we have now further described it and included it in the main text to improve clarity. We have also included a new Table that presents the mean CCI values for the first 400 trials of the motor adaptation process, that is, the early stages of learning. These additional findings reveal that in 16 out of the 22 CCI comparisons the spino-cerebellar model exhibits significantly higher cocontraction than the cerebellar model, whilst only one statistically significant case shows otherwise.

These additional results along with the originally presented findings support the claim that the spino-cerebellar model exhibits higher cocontraction during the development of the internal model, thereby accounting for a behaviour that is more physiologically consistent with the aforementioned biological observations.

We have tried to clarify the aforementioned discussion within the manuscript as follows.

Manuscript lines 282-295, *Results* section *2.4 Spino-cerebellar and cerebellar outcome in muscle space*:

"*Both the spino-cerebellar and cerebellar model exhibited a gradual decrease in joint CCI as motor adaptation evolved (see S15 Fig for CCI evolution). Due to computational constraints, each trajectory repetition was limited to 2000 trials, at the end of which CCI still showed a decreasing trend. The spino-cerebellar model displayed higher CCI values than the cerebellar model in some cases, whilst lower CCI values in others. Consequently, we could not draw definitive conclusions on the final CCI values. Instead, since we used convergence of the kinematic performance as our learning metric, we focused on the CCI values as the kinematic adaptation progressed.*

During the early stages of learning, the spino-cerebellar model exhibited higher overall CCI values than the cerebellar model (see Table 1). Subsequently, we measured the joint CCI once an accurate internal model was developed, as indicated by a stable kinematic performance (see Methods for details on learning convergence metrics). We found that the spino-cerebellar model better reproduced the CCI patterns at the level of the elbow for P1 slow circle and at the level of the shoulder for P2 fast flexion-extension (Fig. 6B). Significantly, the spino-cerebellar model provided higher CCI values at the kinematic convergence point (Fig. 6C) for all P1 and P2 trajectories …"

Manuscript lines 302-306, *Results* section *2.4 Spino-cerebellar and cerebellar outcome in muscle space:*

"*Overall, we highlighted various findings that were consistent for various trajectories with various initial and final positions and speeds. The spino-cerebellar model provided more stable and faster learning with simpler cerebellar synaptic adaptation. Furthermore both models exhibited a gradual reduction in cocontraction as learning progressed; however, the spino-cerebellar model reached learning convergence with higher CCI values and better correlation to the recorded EMG.*"

Manuscript lines 407-418, *Discussion*:

"*There is biological evidence supporting higher cocontraction levels during the early stages of learning, e.g., infants exhibit higher cocontraction during stepping motions compared to adults, and cocontraction is subsequently reduced with practice [Yang, J. F., et al., (1998)]; in the case of the upper limbs, higher cocontraction levels resulting in higher joint stiffness during learning and adaptation to new dynamics have been reported [Franklin, D. W., et al., (2003), Osu, R., et al., (2002)]. These findings suggest that higher cocontraction levels during early learning stages enhance the learning rate and facilitate the acquisition of internal models, which once acquired, enable a gradual reduction of cocontraction [Heald, J. B., et al., (2018)]. In our work, both the spino-cerebellar and the cerebellar model exhibited a gradual decrease in cocontraction levels as the motor adaptation process evolved; thus, both models displayed a biologically plausible behaviour in the broader context of reducing cocontraction through learning. Importantly, the spino-cerebellar model exhibited an overall higher cocontraction level during early stages of learning, and it also provided higher joint CCI values at the learning convergence point, i.e., when an accurate internal model of the upper limb was fully acquired.*"

Manuscript page 16, *Figure 6* and caption*:*

*"**C)** Joint CCI evolution over the 2000-trial motor adaptation process.*"

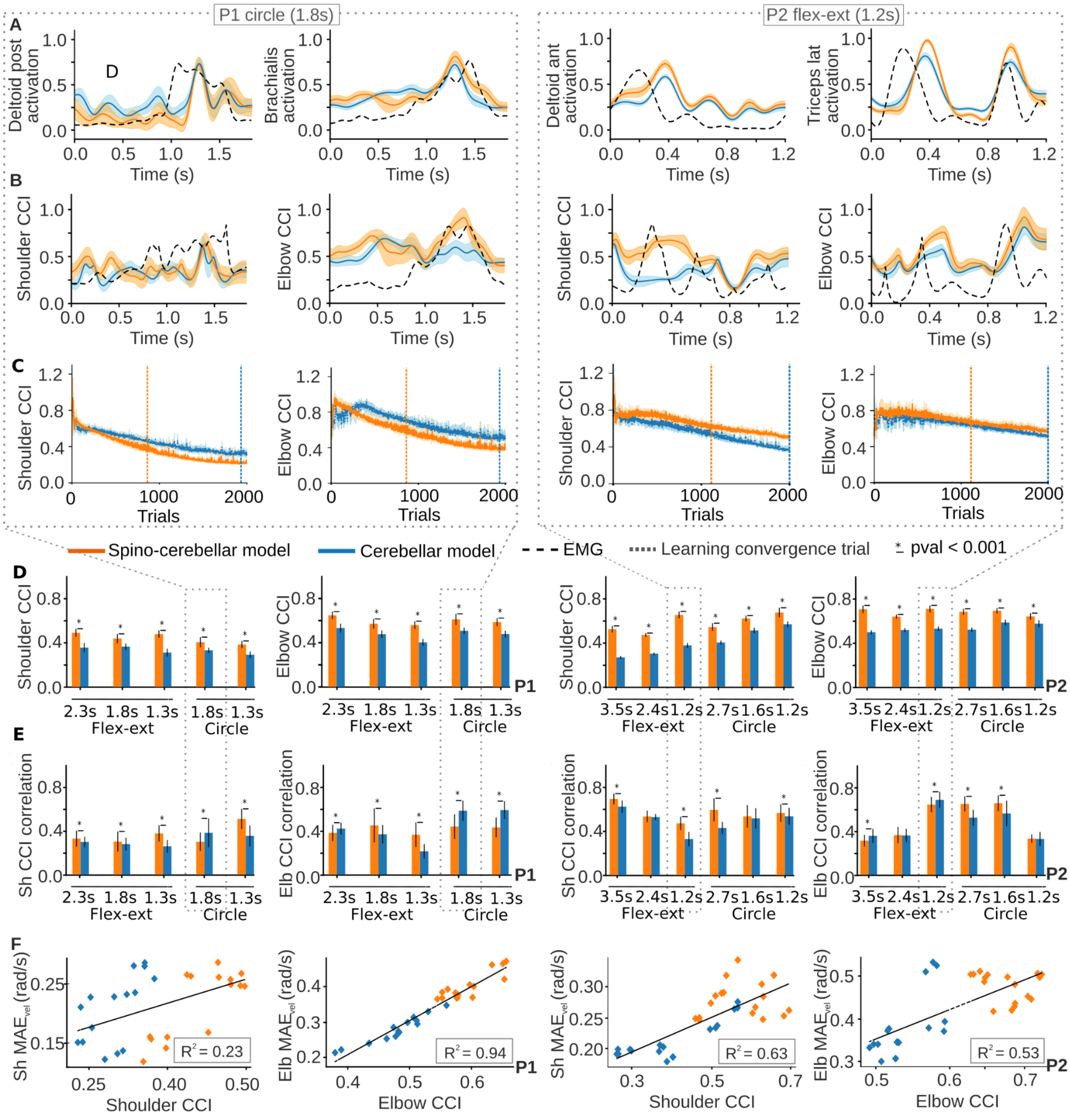

Supplementary Information page 15, new *S15 Figure* and *caption:*

"***Evolution of the joint cocontraction index (CCI) over the motor adaptation process. A), B)*** *Joint CCI for both the spino-cerebellar and cerebellar models during the 2000-trial motor adaptation process for all P1 and P2 trajectories, respectively. Top row shows the shoulder CCI, bottom row displays the elbow CCI."*

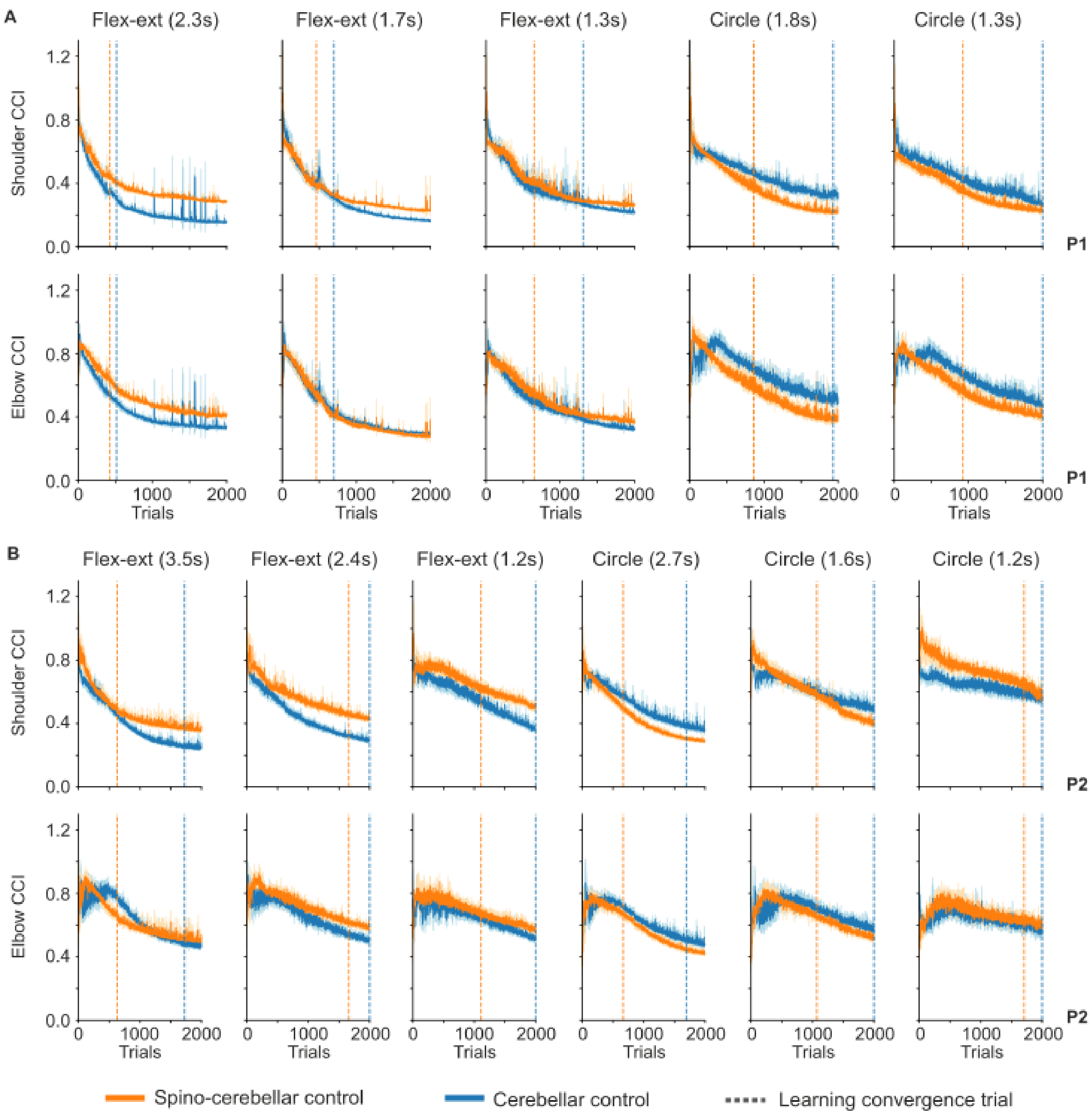

Manuscript page 17, new Table 1 added to *Results* section *2.4 Spino-cerebellar and cerebellar outcome in muscle space*:

"**Table 1.** Joint CCI mean and standard deviation values during the first 400 trials for each P1 and P2 trajectory, for both the spino-cerebellar and cerebellar model."

Trajectory	***Joint***	***Spino-Cerebellar CCI***	***Cerebellar CCI***	***p-value < 0.005***
P1 flex-ext slow	*Shoulder*	*0.58 ± 0.12*	*0.52 ± 0.13*	*
	Elbow	*0.75 ± 0.07*	*0.69 ± 0.10*	*
P1 flex-ext mod	*Shoulder*	*0.56 ± 0.12*	*0.56 ± 0.12*	
	Elbow	*0.71 ± 0.08*	*0.70 ± 0.10*	*

P1 flex-ext fast	*Shoulder*	*0.61 ± 0.10*	*0.60 ± 0.10*	
	Elbow	*0.72 ± 0.06*	*0.70 ± 0.08*	*
P1 circle slow	*Shoulder*	*0.60 ± 0.09*	*0.60 ± 0.07*	
	Elbow	*0.82 ± 0.08*	*0.81 ± 0.09*	*
P1 circle mod	*Shoulder*	*0.54 ± 0.09*	*0.59 ± 0.07*	*
	Elbow	*0.78 ± 0.06*	*0.77 ± 0.06*	*
P2 flex-ext slow	*Shoulder*	*0.69 ± 0.10*	*0.64 ± 0.07*	*
	Elbow	*0.81 ± 0.07*	*0.78 ± 0.08*	*
P2 flex-ext mod	*Shoulder*	*0.73 ± 0.09*	*0.66 ± 0.06*	*
	Elbow	*0.83 ± 0.06*	*0.78 ± 0.07*	*
P2 flex-ext fast	*Shoulder*	*0.76 ± 0.07*	*0.71 ± 0.05*	*
	Elbow	*0.77 ± 0.07*	*0.72 ± 0.07*	*
P2 circle slow	*Shoulder*	*0.68 ± 0.07*	*0.68 ± 0.05*	
	Elbow	*0.73 ± 0.07*	*0.73 ± 0.07*	
P2 circle mod	*Shoulder*	*0.78 ± 0.06*	*0.71 ± 0.05*	*
	Elbow	*0.74 ± 0.09*	*0.70 ± 0.09*	*
P2 circle fast	*Shoulder*	*0.86 ± 0.07*	*0.70 ± 0.04*	*
	Elbow	*0.68 ± 0.08*	*0.65 ± 0.07*	*

From the two spinal-like regulators presented in [*Raphael et al. 2010; J. Neurosci. 30:9431-9444*], the one that incorporates more spinal mechanisms, and thus it is more biologically plausible, facilitated motor learning and yielded results that were more physiologically accurate. In our work we also present two models, and the more biologically plausible model, the spino-cerebellar, similarly facilitates motor learning, supports biological claims, and correlates better with EMG data, thus yielding results that are more physiologically accurate. Therefore, we believe that both our work and [*Raphael et al. 2010; J. Neurosci. 30:9431-9444*] hold equivalent qualitative findings: the spinal cord facilitates learning and produces cocontraction patterns that are more physiologically accurate. This has been clarified within the manuscript as follows.

Manuscript lines 380-383, *Discussion:*

"*Noteworthy, the presence of the SC provided faster motor adaptation, thus assisting cerebellar learning, in line with previous findings on the SC circuitry facilitating motor control of musculoskeletal dynamics [Raphael et al., 2010]. In this regard, a significant finding was the fact that the spino-cerebellar model revealed less complexity at the GC-PC synaptic weight distribution ...* "

Manuscript lines 424-428, *Discussion:*

"*Importantly, the CCI from the spino-cerebellar model also resulted in a better correlation with the CCI patterns from the recorded EMG signals, thus supporting closer biological plausibility than the cerebellar model; incorporating more detailed biological motor control mechanisms into the model increases the level of physiological plausibility in the results [Raphael et al., 2010].*"

- ***Point 1.7.*** *No attempt has been made to see how robust the learned cerebellar solutions are to small changes in the requested trajectory. Robust generalization is an important feature of biological learning that was demonstrated in Tsianos et al. (2014, cited).*

Answer: We agree with the reviewer that robust generalisation is an important aspect of biological learning, comprising cognitive, visual, and motor tasks [*Momennejad, I. (2020). Learning structures: predictive representations, replay, and generalization. Current Opinion in Behavioral Sciences, 32, 155-166; Poggio, T., & Bizzi, E. (2004). Generalization in vision and motor control. Nature, 431(7010), 768-774.*]. When it comes to motor learning, several high-order motor areas in the brain such as the supplementary motor area, the premotor cortex, or the primary motor cortex, contribute to the generalisation of motor skills. Also, brain areas involved in offline motor learning such as the striatum or hippocampus play a role [*Censor, N., Sagi, D., & Cohen, L. G. (2012). Common mechanisms of human perceptual and motor learning. Nature Reviews Neuroscience, 13(9), 658-664.*]. Hence, motor generalisation is usually regarded as a broader brain solution, rather than being exclusive to the cerebellum. Please also note that the cited work by Tsianos et al. (2014) did not include a cerebellar model, but rather "an extremely simple model of the brain" connected to the spinal cord and musculoskeletal system. Thence, it addressed generalisation in the broader context of the central nervous system.

Nonetheless, in previous works, we have studied the cerebellar robustness to small changes:

i) In [*Luque, N. R., et al., (2011). Cerebellar input configuration toward object model abstraction in manipulation tasks. IEEE Transactions on Neural Networks, 22(8), 1321-1328.*] a cerebellar model successfully interpolated a solution for a novel context based on two previously learnt scenarios.

ii) In [*Luque, N. R., et al., (2011). Adaptive cerebellar spiking model embedded in the control loop: Context switching and robustness against noise. International journal of neural systems, 21(05), 385-401.*], a cerebellar model exhibited resilience against noise that affected its input sensory signals.

iii) In [*Abadía, I., et al., (2019). On robot compliance: A cerebellar control approach. IEEE transactions on cybernetics, 51(5), 2476-2489.*] a cerebellar model exhibited robustness to unstructured dynamic changes.

iv) In [*Abadía, I., et al., (2021). A cerebellar-based solution to the nondeterministic time delay problem in robotic control. Science Robotics, 6(58), eabf2756.*] a cerebellar model successfully performed a sequence of motor tasks.

Significantly, all the aforementioned cerebellar models share with the one used in the present work the same five cerebellar neural populations (i.e., mossy fibres, granule cells, Purkinje cells, climbing fibres, and deep cerebellar nuclei), and they all implement the same cerebellar learning mechanism (i.e., spike-timing-dependent plasticity, STDP, at granule cells - Purkinje cells connections), regulated by the balance of long-term potentiation and depression (LTP/LTD) that is driven by the instructive action of climbing fibres. Thus, building upon previous research, the present work aims to investigate the influence of the spinal cord on cerebellar motor learning.

Cerebellar motor research has attracted significant attention in computational modelling approaches. However, for computational reasons or merely due to the pursuit of simplicity, the cerebellum is usually modelled in isolation. We have expanded our previous efforts on understanding cerebellar motor function in the present work to include the spinal cord. We believe that, as emphasised by Tsianos et al. (2014) "any plausible theory of brain function in movement must account for the properties of the spinal circuitry plus musculoskeletal system". In the manuscript, we have made an effort to clarify the scope of our research and highlight the importance of learning generalisation. The following changes have been made in the manuscript.

Manuscript lines 92-97, *Introduction:*

"*Computational models of the cerebellum have been used to study its inner dynamics [Luque, N. R., et al., (2019); Luque, N. R., et al., (2022)], proving the cerebellar motor learning ability and its capacity to adapt to dynamic changes [Luque, N. R., et al., (2011); Luque, N. R., et al., (2011); Abadía, I., et al., (2019); Abadía, I., et al., (2021)]. However, the extensive efforts devoted to cerebellar computational research usually model the cerebellum in isolation. In this work, we build upon and expand previous cerebellar research to include the SC, as theories on the CNS motor function cannot ignore spinal circuitry [Tsianos et al., (2014)].*"

Manuscript lines 590-595, *Methods section 4.1 Cerebellar model*:

"*The cerebellar SNN model was adapted from previous models [Luque, N. R., et al., (2011); Luque, N. R., et al., (2011); Abadía, I., et al., (2019); Abadía, I., et al., (2021)], which have already been used to study cerebellar motor learning, adaptation capabilities, and robustness to dynamic changes. Importantly, the cerebellar model presented here and the ones presented in the aforementioned previous works, all share the same neural populations and learning mechanism. Thus, building upon previous research, the current work focused on the influence of the SC on cerebellar motor learning.*"

Manuscript lines 403-406, *Discussion:*

"*High-order brain functions, such as learning generalisation, have been pointed as key aspects that enable the brain to overcome its physical limitations [Tsianos, G.A., et al., 2014; Poggio, T. et al., 2004]. Here we highlight the interaction between different CNS regions as pivotal for enhancing the brain computational capacity and overcoming its physical constraints.*"

- ***Point 1.8.*** *It is difficult to assess the responses to perturbations (Figs. 7 & S18) in the absence of data about how human subjects respond to similar perturbations. Given the absence of any stretch reflex circuitry in the cerebellar model, it is not surprising that its kinematic responses to perturbations are larger. It isn't clear, however, whether these responses are dominated by such delayed reflex responses or by cocontraction and intrinsic muscle properties that result in corrective forces with zero delay. Not allowing plasticity during any applied perturbations means that we have no data about how the models adapt to changing task conditions, which is likely to be an important role for the cerebellum.*

Answer: We appreciate the feedback provided by the reviewer. The perturbations study was conducted to further support the existing evidence of the spinal cord (SC) involvement in handling external motor perturbations. The experimental setup regarding the subjects recording sessions did not

provide neural activity data, therefore it could not be used to draw any conclusions about the roles of the cerebellum and SC when responding to external perturbations; no inference could have been made on whether the cerebellum or SC would be providing the subjects' main response to the external perturbations. To address this data limitation, we used our computational approach in our study. By including or removing the SC from the control loop, we were able to address the SC influence in responding to perturbations. We have made efforts to clarify this within the manuscript as follows.

Manuscript lines 308-315, *Results section 2.5 The spinal cord increases the robustness against motor perturbations:*

"*The experimental setup used in the previous sections for the subjects recording sessions did not provide neural activity data, thus preventing any conclusions about the roles of the cerebellum and SC in response to external perturbations. It was not possible to determine whether the cerebellum or SC would be the main contributor to the subjects' response to external perturbations. To circumvent this data limitation, we used our computational approach. By including or removing the SC from the control loop we were able to investigate the SC influence in responding to perturbations. To study the response against external perturbations of both the spino-cerebellar and cerebellar models, we used our lab designed benchmark ...* "

Additionally, we have now extended the perturbation benchmark by conducting new experiments to further analyse the separate contributions of the stretch reflex and reciprocal inhibition mechanisms to gain deeper understanding on the SC role in handling motor perturbations. The new set of experiments involved testing two new models against the external perturbations: i) SC equipped only with stretch reflex (SR-cerebellar model); ii) SC equipped only with reciprocal inhibition (RI-cerebellar model). Results showed a prominent contribution played by the stretch reflex mechanisms. For more detailed info on this analysis, please check *Point 2.13* of this review letter.

On the cerebellar side, adaptation to dynamic changes is an important role. In previous modelling research [*Luque, N. R., et al., (2011). Cerebellar input configuration toward object model abstraction in manipulation tasks. IEEE Transactions on Neural Networks, 22(8), 1321-1328.; Abadía, I., et al., (2019). On robot compliance: A cerebellar control approach. IEEE transactions on cybernetics, 51(5), 2476-2489.*] we studied and proved the cerebellum ability to successfully handle external perturbations during motor adaptation under changing task conditions. This current article builds upon those previous works by using an adapted version of the cerebellar model equipped with the very same cerebellar learning mechanisms and neural populations. Hence, our main focus in this work was to examine the SC influence on cerebellar motor control, rather than studying cerebellar motor control itself which was already addressed in previous works. We have now extended this clarification within the manuscript as follows (together with the changes in the *Introduction* and *Methods* section *4.1*, as stated in previous *Point 1.7* of this review letter).

Manuscript lines 336-339, *Results section 2.5:*

"The cerebellar learning capability was disabled in all four cases to prevent adaptation to the perturbations. Note that, in previous research, we studied the cerebellar ability to effectively handle external perturbations during motor adaptation under changing task conditions [Luque et al., 2011, Abadía et al., 2019]. By disabling the cerebellar learning capability, we could specifically focus on the SC contribution."

In terms of intrinsic muscle properties, both the spino-cerebellar and cerebellar cases used identical muscle models with identical mechanical properties. Consequently, the mechanical muscle reaction to perturbations was equal in both cases. This direct equivalence allowed us to concentrate specifically on the contribution of the SC. We have clarified this in the manuscript as follows:

Manuscript lines 434-441, *Discussion:*

"Muscle elasticity has been previously identified as a significant passive contributor in handling perturbations [Burdet, E., et al., (2005)]. Within our framework, the spino-cerebellar and cerebellar models, as well as the SR-cerebellar and RI-cerebellar models, all used identical muscle models with the same mechanical properties. Consequently, the passive muscle contribution to handling perturbations remained equal in all cases. This direct equivalence allowed us to focus on the contribution of the SC and attribute a pivotal role to the SC through the stretch reflex in providing robustness against external perturbations, thus supporting previous physiological and modelling research [Shemmell, J., et al., (2010); Weiler, J., et al., (2019); Stollenmaier, K., et al., (2020); Kistemaker, D.A., et al., (2013)]."

- ***Point 1.9.*** *Fig. 1: The anatomical drawings are too small to see the actions of the modelled muscles and their actions at the shoulder are described incorrectly in the caption – forward motion of the upper arm should be defined as flexion (e.g. anterior deltoid) and backward is extension (posterior deltoid). Their actions are described differently in Methods 4.3 but also incorrectly.*

Answer: We thank the reviewer for pointing out this confusion. We have now included bigger anatomical drawings in Fig. 1, and corrected the description in the figure caption and main text as follows:

Manuscript page 6, *Figure 1* and *caption:*

"***C)*** *Musculoskeletal model. The upper limb model comprised two joints, shoulder and elbow, which were actuated by eight muscles: deltoid anterior and biceps long as shoulder flexors; deltoid posterior and triceps long as shoulder extensors; biceps long, short and brachialis as elbow flexors; triceps long, lateral and medial as elbow extensors.*"

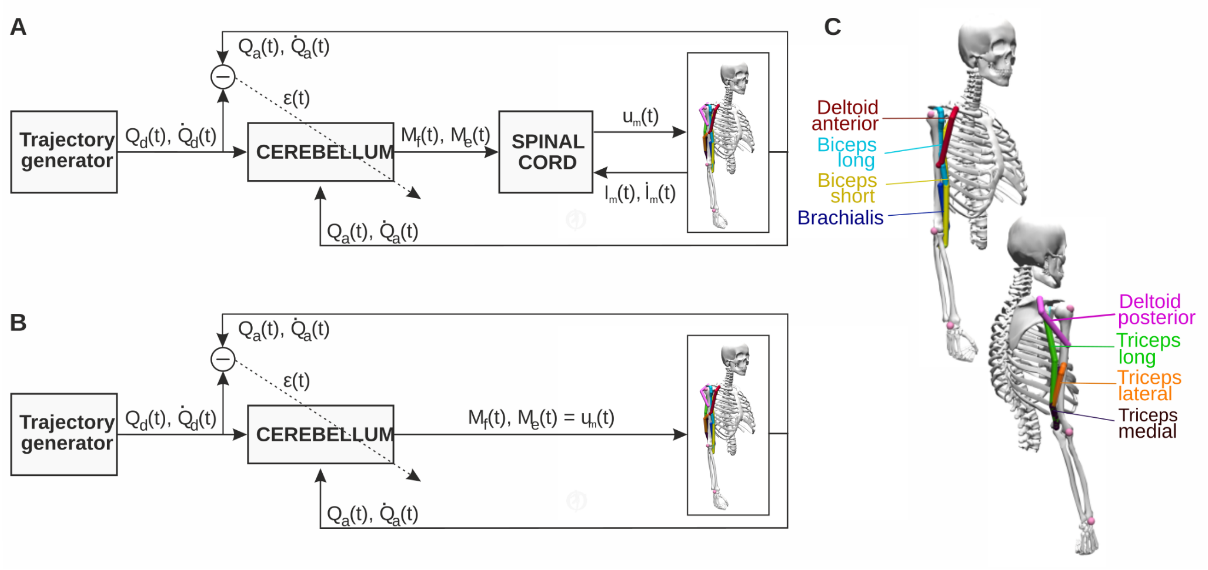

Manuscript lines 662-665, *Methods section 4.3 Musculoskeletal upper limb model:*

“T*he model was actuated by eight Hill-based muscles [Millard, M. et al. (2013)], with the following joint distribution: i) for the shoulder, flexion was carried by the deltoid anterior (DELTant) and the biceps long (BIClong), and extension was conducted by the deltoid posterior (DELTpost) and the triceps long (TRIlong) ...* ”

We thank the reviewer for helping improve our work clarity.

- ***Point 1.10.*** *Fig. 2 legend claims all comparisons in C have p-value <0.001 but text says fast circle comparison is insignificant, as it clearly appears in the figure (which is labeled 1.3s but text says 1.2s). Slow circle (1.8s) doesn't look significant, either. The fluctuating levels of velocity error late in the spino-cerebellar model look like over-training effects. Fig. 3 legend has similar inconsistencies. The authors would do well to use the convention of brackets and asterisks to indicate significance of each comparison rather than summarising these in the figure legends and text.*

Answer: We want to thank the reviewer for helping improve the results clarity. We have modified as suggested Figures 2 and 3 (which are now merged into a single figure, new Figure 2, please see *Point 2.4* of this review letter); statistical significance is now removed from the text and included in the figures using asterisks notation. Statistical significance was obtained performing Welch's t-test [*Welch, Bernard L. "The generalization of 'STUDENT'S'problem when several different population variances are involved." Biometrika 34.1-2 (1947): 28-35.*] on the spino-cerebellar vs cerebellar samples (computed using ttest_ind() function from Python's scipy.stats library [*Virtanen, P., et al., (2020). SciPy 1.0: fundamental algorithms for scientific computing in Python. Nature methods, 17(3), 261-272.*]). This is now specified in Methods section 4.6.1. We also correctly labelled P1's fast circle as 1.3s and P2's fast circle as 1.2s.

Manuscript lines 757-759, *Methods* section *4.6.1 Measuring kinematics performance:*

“We also computed the standard deviation (std) and the T-test p-value between the two models' results with a T-test for the means of two independent samples of values [Welch (1947)] (computed using Python functiopn scipy.stats.ttest_ind [Virtanen, et al. (2020)]).”

Reviewer #2:

Here the authors present a spino-cerebellar model of motor learning with the aim of understanding how the organising principles of the spinal cord can facilitate motor learning.

- ***Point 2.1.*** *This model assumes that the Cerebellum is implementing an inverse model and uses this to generate a desired trajectory, while using the mismatch between the generated trajectory and the desired trajectory as a learning signal. However, Spinocerebellum is generally considered to modulate, not generate, descending motor commands to the spinal command (c.f. Krakauer, John W., et al. "Motor learning." Compr Physiol 9.2 (2019): 613-663., and Cerebellum chapter of Kandel, Eric R., et al., eds. Principles of neural science. Vol. 4. New York: McGraw-hill, 2000.). Furthermore, while there is evidence for inverse models in the Cerebellum, there is arguably more extensive evidence for a forward model (Izawa, Jun, Sarah E. Criscimagna-Hemminger, and Reza Shadmehr. "Cerebellar contributions to reach adaptation and learning sensory consequences of action." Journal of Neuroscience 32.12 (2012): 4230-4239. & Krakauer, John W., et al. "Motor learning." Compr Physiol 9.2 (2019): 613-663.), whereby the Cerebellum attempts to predict sensory consequences of descending motor commands, which in turn provides sensory prediction errors that drives learning (Tseng, Ya-weng, et al. "Sensory prediction errors drive cerebellum-dependent adaptation of reaching." Journal of neurophysiology 98.1 (2007): 54-62.). Given this, it is debatable how well this model reflects what we currently know about Cerebellar computation, and it would be useful if the authors could provide further justifications for the assumptions made in their model.*

Answer: We thank the reviewer for pointing at the mentioned modelling assumptions which, once clarified, will help to better understand and contextualise the contributions of our work. The literature addresses the existence of both inverse and forward models in the cerebellum and, rather than confronting the two model alternatives, there is theoretical, computational, and behavioural experimentation that supports coexistence and complementarity of both approaches [*Wolpert, D. M., et al., (1998). Internal models in the cerebellum. Trends in cognitive sciences, 2(9), 338-347; Ito, M. (2013). Error detection and representation in the olivo-cerebellar system. Frontiers in neural circuits, 7, 1.; Honda, T., et al., (2018). Tandem internal models execute motor learning in the cerebellum. Proceedings of the National Academy of Sciences, 115(28), 7428-7433; Luque, N. R., et al., (2011). Adaptive cerebellar spiking model embedded in the control loop: Context switching and robustness against noise. International journal of neural systems, 21(05), 385-401.; Passot, J. B., et al., (2013). Coupling internal cerebellar models enhances online adaptation and supports offline consolidation in sensorimotor tasks. Frontiers in Computational Neuroscience, 7, 95.*]. However, as suggested by the reviewer, when computationally modelling the cerebellum and using one approach or the other, it is necessary to justify the modelling decisions.

A cerebellar forward model maps control signals and sensory states to predict motor behaviour, whilst an inverse model maps desired motor behaviours to their corresponding motor commands. If the modelling assumption uses the cerebellum as a forward model, then it will modulate the descending motor commands from the motor cortex to correct discrepancies between predicted and actual motor behaviours. In this case, the descending motor commands would already incorporate the arm-plant dynamics, and the cerebellum will only adjust deviations from those dynamics learnt in higher brain areas. On the other hand, when the cerebellum operates as an inverse model, it acquires the entire arm-plant dynamics.

The spinal cord (SC), with its direct action over muscles, significantly modifies the arm-plant dynamics. If the implemented cerebellar model was a forward model, it might improve tracking

performance, but it would hinder evaluation of the influence of the spinal cord on cerebellar learning. The SC modification of the arm-plant dynamics would affect the cerebellar learning but it would also impact the descending motor commands, thus making it difficult to assess the motor action from the cerebellum and from the SC separately, i.e., comparison between our spino-cerebellar vs. cerebellar cases would be hampered. Using the cerebellum as an inverse model puts our implementation in a worst-case scenario in which the cerebellum must abstract the entire arm-plant dynamics to achieve the desired trajectory, but at the same time, this configuration allows direct assessment of the effects of the SC on cerebellar motor learning. By isolating the role of cerebellum in motor control, we are able to study how the SC influences the cerebellum ability to learn and adjust motor commands, i.e., comparison between our spino-cerebellar vs. cerebellar cases is straightforward.

To address this and other modelling assumptions we have added a new subsection called "***Cerebellum and spinal cord modelling assumptions***", added to the manuscript *Discussion* section, lines 477-560. The full new subsection can be found at the end of this review letter for simplicity, please refer to paragraphs 2 and 3 which address the modelling assumptions discussed above.

We appreciate the feedback and hope to have justified the modelling assumptions.

- ***Point 2.2.*** *It could also be argued that certain findings described here are unsurprising: it seems self-evident that EMG/CCI data from human subjects is better matched by a model with reflex loops that are known to heavily shape coordinated muscle activity in natural movement, and we would fully expect greater robustness to perturbations when co-contraction mechanisms are hard wired by reciprocal inhibition and reflex loops rather than allowing the cerebellar model to try and learn them. Nonetheless these observations do provide useful emphasis on the importance of including such mechanisms when building models of sensorimotor loop. The significance of the paper would be improved if a deeper analysis was provided for the more notable/novel findings regarding improved motor learning rates and simpler solutions in the synaptic weight space when a spinal cord interface is included.*

Answer: We thank the reviewer for helping us to better focus the article contributions. The purpose of correlating the spino-cerebellar and cerebellar cases with the EMG data was to validate the model implementation. More concretely, we aimed to demonstrate that the spino-cerebellar case performs the motor tasks in a more biologically plausible manner, closely resembling the natural movements performed by both subjects. As a result, these findings supported the improvement in motor learning when the SC was included in the loop, both at kinematic and synaptic levels. We have made the following modifications on the manuscript to better clarify these points and further highlight the importance of detailed modelling when building models of the sensorimotor loops:

Manuscript lines 424-428, *Discussion*:

"*Importantly, the CCI from the spino-cerebellar model also resulted in a better correlation with the CCI patterns from the recorded EMG signals, thus supporting closer biological plausibility than the cerebellar model; incorporating more detailed biological motor control mechanisms into the model increases the level of physiological plausibility in the results [Raphael et al., 2010].*"

Additionally, to further deepen the findings relating to the synaptic weight space, we have now included another metric quantifying the % of granule cell - Purkinje cell connections (GC-PC) required to perform the motor tasks by both spino-cerebellar and cerebellar cases. For that we measured the % of GC-PC synapses that modified their initial weight as the motor adaptation process evolved. Results show how the spino-cerebellar case makes use of fewer GC-PC connections for all P1 & P2 motor tasks; the SC allows reducing the GC neurons required for execution of the motor tasks and, consequently, there is more "computational space" at the CG layer.

GCs are the most numerous neuron type in the whole brain, and the granular layer can be seen as a reservoir computing mechanism increasing the dimensionality of the received sensorimotor inputs. The fact that the SC simplifies learning at the granular layer might be key for a more efficient use of cerebellar resources and increasing its computational capacity. The interaction between different CNS regions is key for increasing brain computational capacity and circumventing its physical limitations. The following modifications have been made to the manuscript to further discuss the implications of the simpler solutions in the synaptic weight space when the SC is in the loop:

Manuscript lines 252-258, *Results section 2.3 The spinal cord simplifies cerebellar synaptic adaptation at GC-PC:*

"To deepen in the finding of the SC simplification of the cerebellar synaptic solutions, we analysed the amount of GC neurons required by the spino-cerebellar and cerebellar models. We measured the percentage of GC-PC synapses that experienced a modification of their initial weight as motor adaptation progressed, thus providing a measurement of how many GC-PC connections were effectively involved in motor learning (Fig. 4E and F). Results showed that the spino-cerebellar model made use of fewer GC-PC connections for all P1 and P2 motor tasks. Thus, the SC allowed for a reduction of the GC neurons required for accurate execution of the motor tasks."

Manuscript lines 382-406, *Discussion*:

"*In this regard, a significant finding was the fact that the spino-cerebellar model revealed less complexity at the GC-PC synaptic weight distribution and it required fewer GC neurons for accurate execution of the motor tasks: the SC led to the formation of less specialised GC-PC synapses, thus freeing up computational resources within the cerebellum. The cerebellar granular layer can be compared to a reservoir computing mechanism [Yamazaki, T., & Tanaka, S. (2007). The cerebellum as a liquid state machine. Neural Networks, 20(3), 290-297.][Tokuda, K., Fujiwara, N., Sudo, A., & Katori, Y. (2021). Chaos may enhance expressivity in cerebellar granular layer. Neural Networks, 136, 72-86.], wherein the cerebellum increases the dimensionality of the sensorimotor inputs that it receives. The ability of the SC to facilitate a more efficient use of GC neurons (the most numerous neuron type in the mammalian brain [Consalez, G. G., et al., (2021). Origins, development, and compartmentation of the granule cells of the cerebellum. Frontiers in neural circuits, 88.]) can be compared to increasing the number of units in reservoir computing. Increasing the size of the reservoir enhances its computational power and expands its memory capacity [Lukoševičius, M. (2012). A practical guide to applying echo state networks. In Neural Networks: Tricks of the Trade: Second Edition (pp. 659-686). Berlin, Heidelberg: Springer Berlin Heidelberg.][Cucchi, M., et al., (2022). Hands-on reservoir computing: a tutorial for practical implementation. Neuromorphic Computing and Engineering.]. A larger size increases the degrees of freedom of the reservoir response, allowing it to capture more complex dynamics. A larger size also allows the reservoir to store greater amounts of values . Thus, by facilitating a more efficient use of*

cerebellar resources, the SC enables an increase in the computational capacity of the cerebellum, hence overcoming its physical limitations. To the best of our knowledge, it is the first time that a computational model highlights and weighs the influence of the SC in facilitating cerebellar learning.

Direct regulation of muscle activity by the SC has here been found to facilitate the cerebellar acquisition of the upper limb inverse dynamics. Indeed, the body plant dynamics to be learnt by higher brain areas, might be simplified by the SC taking over lower level and faster control primitives, such as the SC potential role in gravity compensation [Klauer, C., et al., 2014; Ritzman, R., et al., 2017]. Thus, the SC performance in muscle space may lighten other operations of the sensorimotor process, occurring at a higher level such as the cerebellum's contribution in compensating interaction torques in joint space [Bastian, A.J., et al., 1996], or in shaping spatiotemporal muscle synergies rather than generating specific complex muscle patterns [Berger, D.J., 2020]. High-order brain functions, such as learning generalisation, have been pointed as key aspects that enable the brain to overcome its physical limitations [Tsianos, G.A., et al., 2014; Poggio, T. et al., 2004]. Here we highlight the interaction between different CNS regions as pivotal for enhancing the brain computational capacity and overcoming its physical constraints."

Figure 4 has been also modified to include the new results. Please also see *Points 2.9* & *2.10* of this review letter for a further discussion on the data displayed on Fig. 4.

Manuscript page 12, *Figure 4* and *caption*:

"***Spino-cerebellar and cerebellar synaptic adaptation**. **A)**, **B)** Synaptic weights at granule cell - Purkinje cell (GC-PC) synapses after 200 and 2000 trials, respectively, for both models performing P1's 1.8s circle trajectory. The heat map represents the normalised GC-PC synaptic weights, which could range from 0.0 to 15.0 nS. **C)**, **D)** Evolution of the synaptic entropy at the GC-PC synapses over the 2000-trial motor adaptation process, for all P1 and P2 trajectories, respectively. The higher the entropy, the more complex the GC-PC synaptic distribution (i.e., higher heterogeneity in the synaptic weights of the GC-PC synapses). **E)**, **F)** Percentage of GC-PC synapses that experienced a modification of their initial weight as motor adaptation progressed for all P1 and P2 trajectories; i.e., amount of GC-PC synapses required by both the spino-cerebellar and cerebellar models to succeed in the motor adaptation process.*"

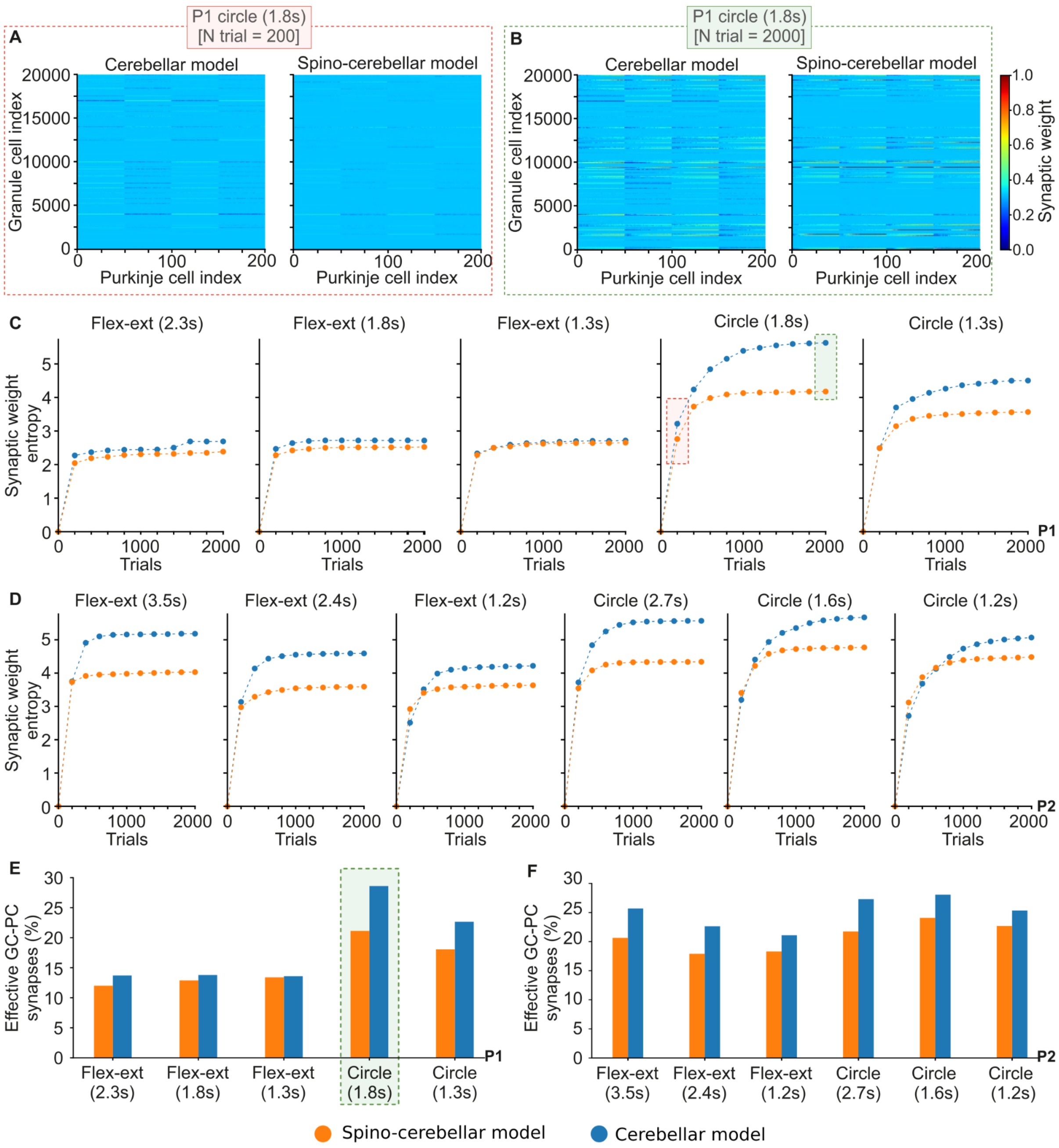

We hope these modifications will provide greater clarity and better focus for the article contributions.

Additional comments:

i) ***Point 2.3.*** *Was any optimisation of network parameters required? If so, how was this performed?*

Answer: We performed an exhaustive search on the STDP parameters that govern the learning dynamics of both the spino-cerebellar and cerebellar cases, as described in [*Luque, N. R., et al., (2019). Spike burst-pause dynamics of Purkinje cells regulate sensorimotor adaptation. PLoS computational biology, 15(3), e1006298.; Luque, N. R., et al., (2022). Computational epidemiology study of homeostatic compensation during sensorimotor aging. Neural Networks, 146, 316-333.; Luque, N. R., et al., (2022). Electrical coupling regulated by GABAergic nucleo-olivary afferent fibres facilitates cerebellar sensory-motor*

sensory–motor adaptation. Neural Networks, 155, 422-438.], since these parameters had the greatest impact on the spino-cerebellar and cerebellar outputs (LTP increment was given by parameter α = 0.006nS, and LTD decrement was influenced by β = -0.003nS, please see *Methods* section *4.1* for the mathematical description of the LTP/LTD mechanism, together with Table 1 for the network topology). Our goal was not to optimise parameters specifically for the spino-cerebellar and cerebellar cases based on behavioural data, but rather to identify a common set of parameters that, when applied to both cases, would enable a fair comparison between them. The selected parameters were chosen to cover the full working range of the different motor tasks. To ensure a fair comparison of the performance of the spino-cerebellar and cerebellar cases, we used a common set of network parameters for both cases. Our focus was to provide parameters that allowed for the full range of motor tasks, rather than optimising them for specific use cases.

Manuscript page 26, footnote added to *"Table 2. Cerebellar neural topology"*, in *Methods* section *4.1 Cerebellar model*:

"*We performed an exhaustive search on the STDP parameters that govern the learning dynamics of both the spino-cerebellar and cerebellar models, as described in [Luque, N. R., et al., (2019).; Luque, N. R., et al., (2022)a.; Luque, N. R., et al., (2022)b.], since these parameters had the greatest impact on the models output commands. The selected parameters were chosen to cover the full working range of the different motor tasks. To ensure a fair comparison of the performance of the spino-cerebellar and cerebellar models, we used a common set of network parameters for both cases.*"

ii) ***Point 2.4.*** *Having 2 separate figures (fig 2 and 3) for each subject seems excessive. Can you combine these two into a single figure?*

Answer: Thank you for helping improve the conciseness of the manuscript. Figures 2 and 3 are now merged into a single figure (new Fig 2).

Manuscript page 8, *Figure 2* and *caption:*

*"**Spino-cerebellar and cerebellar models kinematic performance for the lab recorded scenario. A)** Position and velocity mean absolute error (MAE) over the 3 repetitions of the 2000-trial motor adaptation process; and joint kinematics of the 3 repetitions last 200 trials (mean and standard deviation) for both the spino-cerebellar and cerebellar models performing P1's slow circle trajectory (1.8s), and **B)** P2's fast flexion-extension (1.2s). **C)** Mean and standard deviation of the position and velocity MAE (last 200 trials of the 3 trajectory repetitions) for all P1 recorded trajectories, and **D)** for all P2 recorded trajectories."*

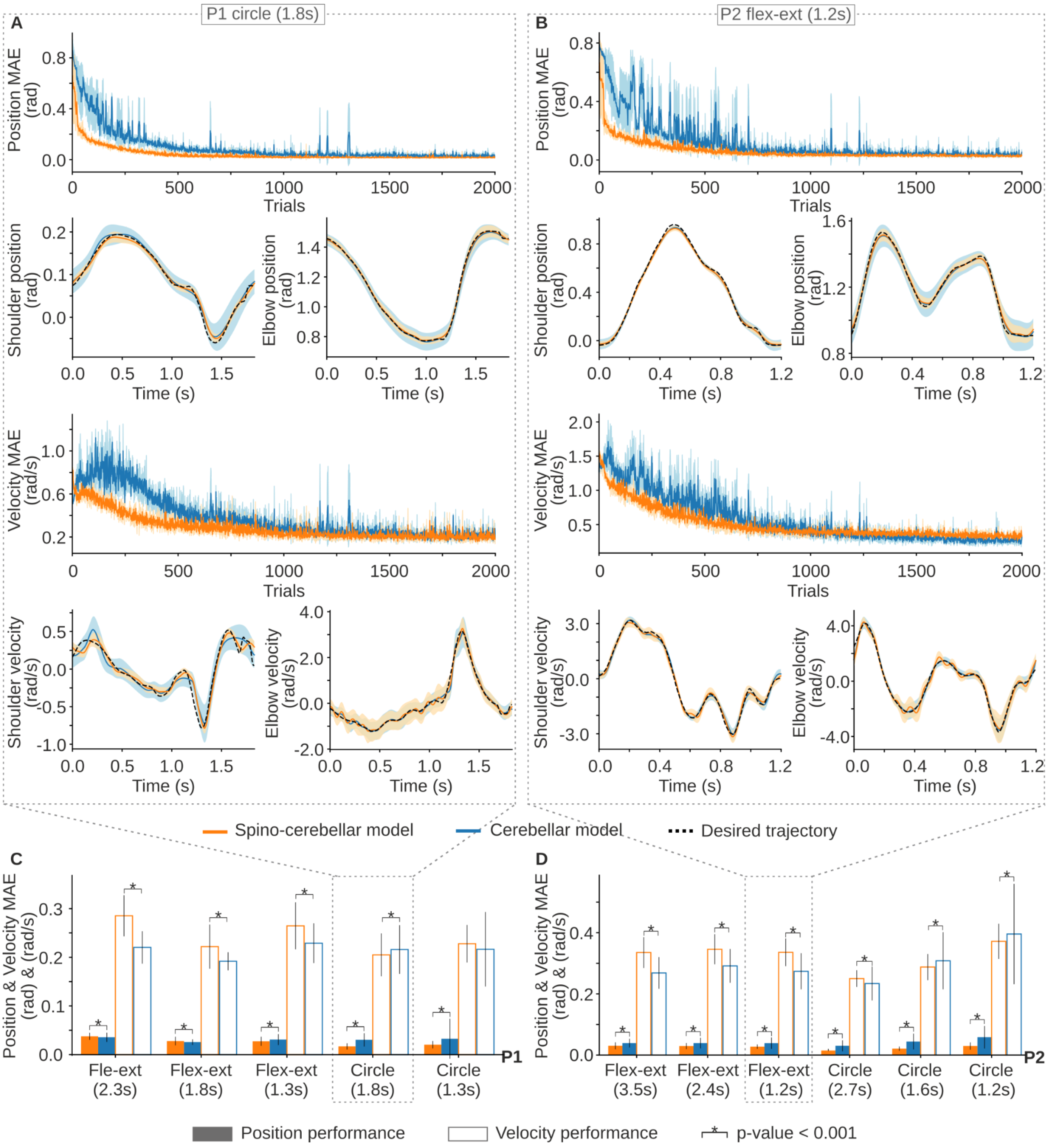

iii) ***Point 2.5.*** *Why 0.1 rad and 0.5 rad/s for convergence targets?*

Answer: To measure learning speed, we used a MAE target that was common to all motor tasks whilst taking into account the diversity of final MAE values amongst the different motor tasks. The final MAE mean of all motor tasks (including both spino-cerebellar and cerebellar cases) was 0.03 rad for position, and 0.27 rad/s for velocity. To set the learning speed targets, we doubled these mean values and rounded them to the nearest tenth, resulting in 0.1 rad for position and 0.5 rad/s for velocity. We have clarified this within the manuscript as follows.

Manuscript lines 776-783, *Methods section 4.6.2 Measuring learning performance*:

"Additionally, we assessed the learning speed of the two models by considering the number of trials required to reach a target MAEpos of 0.1rad and a target MAEvel of 0.5rad/s. We defined the learning speed metric as 1 over this number of trials (N−1 trials). The target values (0.1 rad and 0.5 rad/s) were such that they provided a common measure for all motor tasks whilst also taking into account the diversity of final MAE values amongst the different motor tasks. The final mean MAE of all motor tasks (including both spino-cerebellar and cerebellar models) was 0.03 rad for position, and 0.27 rad/s for velocity. To set the learning speed targets, we doubled these mean values and rounded them to the nearest tenth, resulting in 0.1 rad for position and 0.5 rad/s for velocity."

We hope this explanation provides a clearer description on how the learning speed was calculated. Thank you again for your comment and feedback.

iv) ***Point 2.6.*** *Panel A in all of figures 2-4 should ideally have a shaded region around the mean line to show standard deviation of the learning curves.*

Answer: We thank the reviewer for helping improve the clarity of the results. The mentioned figures have been modified as suggested. In Figure 3 panel A, the learning curve of only one of the repetitions of the motor adaptation process is displayed (no std) as an example of how learning convergence and learning speed metrics are computed over the individual learning curves; i.e., the learning metrics were computed for each of the 3 repetitions of the motor adaptation process, and then used to compute the mean and std (shown in Fig.3B and C).

Manuscript page 10, *Figure 3* caption:

"Only one repetition of the motor adaptation process is displayed."

v) ***Point 2.7.*** *"To study learning convergence we applied control charts on the MAE data to determine the number of trials required to achieve a stable performance [39]." The definition of learning convergence could do with a more thorough description here (just one extra sentence better describing how to is calculated).*

Answer: We want to thank the reviewer for bringing this to our attention. As suggested, we have now further described how learning convergence is calculated:

Manuscript lines 206-211, *Results section 2.2 The spinal cord improves cerebellar learning convergence and speed:*

"To study learning convergence, we applied control charts on the MAE data to determine the number of trials required to achieve a stable performance [Roberts, S.W. (1996)]. We computed the MAE mean (μ) and standard deviation (σ) using a temporal sliding window with a sample size of 200 trials and defined different MAE limits relating μ and σ (e.g., limit 1 = MAE ∈ [μ - σ , μ + σ]). We then measured the percentage of trials with a MAE value within each limit (see Methods)."

We hope this explanation provides greater clarity. Thank you again for your comment and feedback

vi) *Point 2.8.* *Figure 4B-C should have error bars or std dev bars and statistical tests should be performed to confirm learning convergence and learning rates are significantly different for Cb-SC vs Cb-only models.*

Answer: We thank the reviewer for helping improve the presentation of the results. Fig 4B-C (new Figure 3B-C) has been modified as suggested. New Figure 3, page 10:

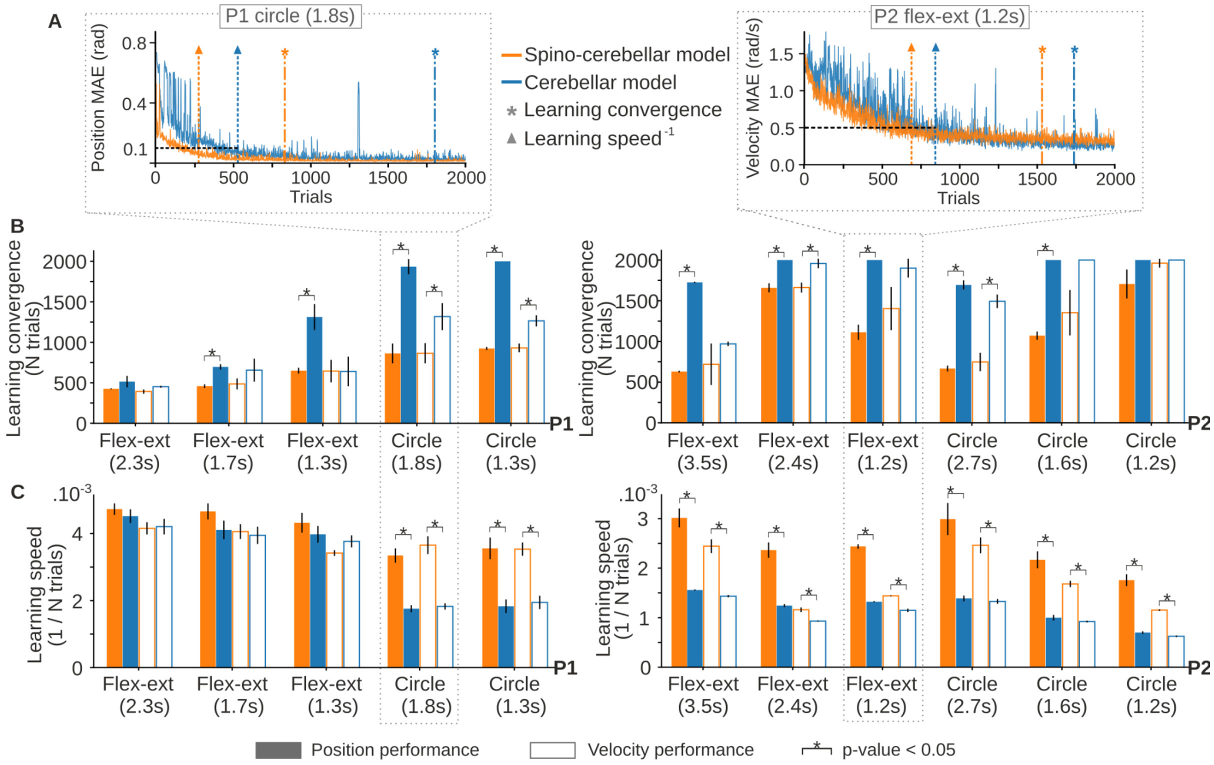

In the previous version these results showed the measurements provided for one 2000-trial repetition, apologies for not realising before. Now the mean and std of the three 2000-trial repetitions are displayed, and the values shown in the main text have been updated as well (section *2.2 The spinal cord improves cerebellar learning convergence and speed*, lines 216-223).

vii) *Point 2.9.* *Figure 5C,D could be more effectively visualised as % change in synaptic entropy relative to entropy at trial 0, and accompanied with a second plot with trials on the x axis, and difference between synaptic entropy in the Cb-SC vs Cb-only models on the y-axis. Then you can bin the Flex-ext and Circle tasks together into a one % change plot and one entropy difference plot for each task.*

Answer: Thank you for addressing this Figure which presents some of the core results of our work (original Figure 5 now corresponds to Figure 4 in the updated manuscript). We apologise for any confusion that may have arisen due to insufficient information about the initial state of the synaptic weight distribution at trial 0, which we aim to clarify now. Typically, synaptic weights that undergo plasticity are initialised with random values. However, for the sake of clearly comparing the CB-SC vs CB-only cases, the results presented in this section correspond to a homogeneous initialization of the GC-PC synaptic weights for all motor tasks: i.e., at trial 0 all GC-PC synapses started with the same synaptic weight (4.8 nS). The previous sections include results from three repetitions of the motor adaptation process; each repetition consisted of 2000 trials. Two of those repetitions were initialised with random weights whilst one was homogeneously initialised, serving as our synaptic

adaptation benchmark. Since we initialised the GC-PC synaptic weights with a homogeneous distribution, the synaptic entropy at trial 0 was equal to 0.0. Consequently, all dashed lines in Fig. 4C, D start from 0.0 and increase as trials progress. We have now clarified this within the manuscript and figure caption.

Manuscript lines 227-236, *Results section 2.3 The spinal cord simplifies cerebellar synaptic adaptation at GC-PC:*

"The effect of the SC on cerebellar learning, already checked in terms of motor performance in the previous section, must leave its trace at the level of cerebellar synaptic adaptation. To conduct a direct comparison between the synaptic adaptation of the spino-cerebellar and cerebellar models, it is necessary to establish a common synaptic foundation. Thus, in one of the three repetitions of the 2000-trial motor adaptation process for each motor task, the synaptic weights between GCs and PCs were homogeneously initialised; i.e., at trial 0 all GC-PC synapses in both the spino-cerebellar and cerebellar models started with the same synaptic weight (4.8 nS). This homogeneously-initialised, 2000-trial repetition for the different P1 and P2 motor tasks, served as our benchmark for synaptic adaptation. The common starting point concerning the synaptic weight distribution allowed for a fair comparison of the synaptic evolution between the two models".

Manuscript page 26*, Methods* section *4.1, Table 2 caption:*

*"**Table 2.** Cerebellar neural topology. Dashed entries stand for not applicable. Each GC-PC synapse was randomly initialised within the range [4.3, 5.2nS], except for one of the three repetitions of the 2000-trial motor adaptation process for each motor task. In that specific repetition, all GC-PC synapses were homogeneously initialised with 4.8nS."*

Manuscript lines 787-790*, Methods* section *4.6.3 Measuring cerebellar synaptic adaptation:*

"*To conduct a direct comparison between the synaptic adaptation of both the spino-cerebellar and cerebellar models, the GC-PC synaptic weights were homogeneously initialised in one of the three repetitions of the motor adaptation process for each motor task; thus providing a common synaptic starting point that allowed studying the differences at the synaptic level.*"

Also regarding these results at the synaptic weight space, we have conducted a new analysis measuring the % of GC-PC connections that are effectively used by the CB-SC and CB-only cases; i.e., the GC-PC synapses that modify their initial weight through the motor adaptation process. Results show how the CB-SC case requires fewer synapses to perform the motor tasks: the % of effectively used synapses is smaller for all P1 & P2 motor tasks. Please see *Point 2.2* of this review letter for a further discussion on the implications of this finding. We have included these new results in the new Fig. 4, which we hope will allow a more effective visualisation of the results.

Manuscript lines 805-807*, Methods* section *4.6.3 Measuring cerebellar synaptic adaptation:*

"*To measure the number of GC neurons required by the spino-cerebellar and cerebellar models to successfully adapt to each motor task, we measured the percentage of GC-PC synapses that, by the end of the 2000 trials, had experienced a modification of their initial weight (set to 4.8nS, see Table 2).*"

viii) ***Point 2.10.*** *Figure 5A,B could be used for a more interesting analyses, as it is not particularly surprising that the synaptic weights change over trials. What about checking if Cb-SC and Cb-only models converge to the same synaptic solutions every time, and if they converge to the same solutions as each other?*

Answer: We thank the reviewer for bringing this up. Original Figure 5 now corresponds to Figure 4. Figures 4A and B show the GC-PC synaptic weight distribution for both CB-SC and CB-only cases at trial 200 (early learning stage) and trial 2000 (end of the motor adaptation experimental setup), respectively. We understand that the synaptic change may not be surprising, but we believe that these plots provide the reader with an insight about what is actually happening in the cerebellum at a synaptic level. These figures also help contextualise the core results of this section shown in Figure 4C and D (as discussed in *Point 2.9* of this review letter).

We measured the effect of the spinal cord on cerebellar learning at the synaptic level by computing the entropy of the GC-PC synaptic weight distribution every 200 trials, i.e., comparing the complexity of CB-SC vs. CB-only synaptic solutions over time. The snapshot in Fig. 4A shows the first synaptic distribution recorded at 200 trials, whereas in B it shows the last record taken at 2000 trials. Thus, Fig. 4A and B intend to provide a visual representation of what we are measuring the entropy of in Fig. 4C and D. New Fig.4 has been modified to better link panels A, B and C, and also the manuscript has been modified to further clarify this as follows:

Manuscript lines 239-243, *Results* section *2.3 The spinal cord simplifies cerebellar synaptic adaptation at GC-PC:*

"*We then measured the entropy of the GC-PC synaptic weight distributions to quantify the synaptic complexity of both models: the higher the entropy, the more complex the synaptic weight distribution, i.e., higher heterogeneity of synaptic weights at the GC-PC population. The synaptic entropy metric can be grasped as measuring the complexity of the synaptic patterns displayed in Fig.4A and B, and the rest of the patterns recorded every 200 trials describing the full motor adaptation process.*"

The entropy plots then illustrate that CB-SC and CB-only do not converge to the same synaptic solution as each other; the SC simplifies the weight distribution for all motor tasks, thus the synaptic solutions cannot be the same. Our apologies for any confusion, we have now tried to clarify this in the manuscript as follows:

Manuscript lines 258-261, *Results* section *2.3 The spinal cord simplifies cerebellar synaptic adaptation at GC-PC:*

"The common synaptic starting point together with the synaptic entropy evolution and the amount of neurons involved in the motor adaptation process, support the divergence in the synaptic solutions acquired by the spino-cerebellar and cerebellar models, and the SC influence in facilitating cerebellar learning at the synaptic level."

ix) ***Point 2.11.*** *Figures should more clearly label which plots correspond to which subjects.*

Answer: Agreed and corrected. Figures 2, 3, 4, and 5, and 6 have been corrected to include "P1" and "P2" labels, which will help to unambiguously identify the corresponding subject.

x) ***Point 2.12.*** *Figure 6A/B could be more informative if the y-axis is the absolute difference between the muscle activation signal and the EMG data.*

Answer: We appreciate the feedback provided by the reviewer. Figure 6A/B intends to display similarities in the muscle activation patterns provided by the spino-cerebellar and cerebellar models and EMG data. We focused on muscle activity patterns (correlation measurement) rather than measuring amplitude differences point by point since, as stated in the manuscript sections *2.4* and *4.6.5*, it is not straightforward to draw conclusions from a direct comparison between the muscle activity of the simulated models and recorded EMG, mainly due to:

i) Scaling issues as EMG are difficult to normalise and subject to measurement errors as highlighted by [*Hicks, J. L., et al. "Is my model good enough? Best practices for verification and validation of musculoskeletal models and simulations of movement." Journal of biomechanical engineering 137.2 (2015): 020905.*]. Whilst simulated muscle activation signals are commonly compared to EMG processed envelopes [*Buchanan, T. S., et al. "Neuromusculoskeletal modeling: estimation of muscle forces and joint moments and movements from measurements of neural command." Journal of applied biomechanics 20.4 (2004): 367-395.*], Hicks, J. L., et al. recommend to check for similar salient features and trends. Thus, correlation coefficients are often used as a metric to validate models in this manner [*Wang, R., et al. "Clarify sit-to-stand muscle synergy and tension changes in subacute stroke rehabilitation by musculoskeletal modeling." Frontiers in Systems Neuroscience 16 (2022): 28., Michaud, F., et al. "A fair and EMG-validated comparison of recruitment criteria, musculotendon models and muscle coordination strategies, for the inverse-dynamics based optimization of muscle forces during gait." Journal of neuroengineering and rehabilitation 18 (2021): 1-15., Afschrift, M., et al. "The effect of muscle weakness on the capability gap during gross motor function: a simulation study supporting design criteria for exoskeletons of the lower limb." Biomedical engineering online 13.1 (2014): 1-15.*].

ii) Our musculoskeletal model is a simplification of the human upper limb.

To overcome this constraint, we used correlation between activation signals and EMG envelopes, and complemented the analysis with the more comprehensive metric provided by the joint cocontraction index (CCI). This has now been further clarified within the manuscript as follows:

Manuscript lines 822-827, *Methods* section *4.6.5 Measuring muscle space performance:*

"We also evaluated performance in the muscle space using the lab recorded benchmark. Activation signals from models are commonly compared to EMG envelopes, but such comparisons are generally difficult to achieve due to scaling issues that hinder a direct analogy between the model and the real muscle dynamics; EMG signals are difficult to normalise and subject to measurement errors [Hicks et al., (2015)]. Besides, our musculoskeletal model is a simplification of the human upper limb, thus further hindering direct comparison of recorded EMG and the models muscle activation. To overcome this issue, we followed a more comprehensive approach by computing the correlation between activation signals and EMG envelopes; a commonly adopted solution to the aforementioned limitations [Wang, R., et al. (2022); Michaud, F., et al. (2021); Afschrift, M., et al. (2014)]."

xi) *Point 2.13.* *How dependent is perturbation stability on the reflex vs reciprocal inhibition parts of the SC module?*

Answer: To provide a deeper insight into the SC contribution in handling perturbations, we have now included a new study that individually assessed the weight of the stretch reflex and reciprocal inhibition mechanisms in the overall SC contribution. We conducted the same set of external perturbations (increasing the number of perturbed-trials up to 50 for all cases) and distinguished between two additional cases: i) SC equipped only with stretch reflex (SR-cerebellar model), ii) SC equipped only with reciprocal inhibition (RI-cerebellar model). To focus on the contribution of the two SC mechanisms and be able to conduct a direct comparison, for the new SR-cerebellar and RI-cerebellar cases, we set the cerebellar synaptic weights to those obtained by the spino-cerebellar model and then disabled cerebellar adaptation; i.e., we equipped the cerebellar network with the synaptic solution acquired by the cerebellum after motor adaptation when the SC model was fully equipped, thus preventing the cerebellum from learning how to compensate for the deficiencies induced by the lack of spinal mechanisms. These results, along with the previously provided baseline results for the fully-equipped SC (spino-cerebellar model) and non-SC (cerebellar model), now enable us to assess the contribution of each spinal mechanism in handling external perturbations. The deviation results of the four models were compared using a Kruskal-Wallis H-test (Python function scipy.stats.kruskal) to assess the overall difference amongst the four groups, followed by a Dunn test (Python function scikit-posthocs.posthoc_dunn) to conduct pairwise tests.

The updated Figure 7B and D demonstrate that the SR-cerebellar model exhibits smaller position and velocity deviation for the majority of the eight perturbation types applied during the moderate flexion-extension trajectory compared to the RI-cerebellar model. In comparison to the fully equipped spino-cerebellar model, the SR-cerebellar model exhibits similar deviation for most of the perturbations, whilst the RI-cerebellar model exhibits an overall larger deviation. Overall, the spino-cerebellar and the SR-cerebellar models exhibit the smallest average deviations for all the trajectories, as observed in Figures 7C and 7E. This indicates that the SR-cerebellar model exhibits greater robustness compared to the RI-cerebellar in terms of both position and velocity deviation across all trajectories.

We also compared the joint cocontraction index (CCI) values of the four models, provided during performance of the three trajectories without induced perturbations. The SR-cerebellar exhibited the highest CCI values, followed by the spino-cerebellar, and then the RI-cerebellar and cerebellar models. It was evident that the stretch reflex increased cocontraction, whilst RI decreased cocontraction. Thus, we observe a relationship between CCI and robustness against perturbation, with models having higher CCI values resulting in smaller trajectory deviations.

As discussed in the first version of the manuscript, cocontraction plays an important role in motor stability [*Spoelstra, J., et al., Cerebellar learning of accurate predictive control for fast-reaching movements. Biological Cybernetics, 82(4):321–333, March 2000, Latash, M.L. Muscle coactivation: definitions, mechanisms, and functions. Journal of Neurophysiology, 120(1):88–104, July 2018*]. Our perturbation study reveals that the fully-equipped SC and stretch-only models display the highest robustness against perturbation, accompanied by significant levels of cocontraction, compared to the RI-only and cerebellar-only models. This highlights the major role of the stretch reflex and cocontraction in handling perturbations. Our findings are consistent with previous physiological and modelling studies *[Shemmell, J., et al., "Stretch sensitive reflexes as an adaptive mechanism for maintaining limb stability." Clinical Neurophysiology 121.10 (2010): 1680-1689; Weiler, J.,et al., Spinal stretch reflexes support efficient hand control. Nature Neuroscience, 22(4):529–533, February 2019; Stollenmaier, K., et al., Predicting*

perturbed human arm movements in a neuro-musculoskeletal model to investigate the muscular force response. Frontiers in Bioengineering and Biotechnology, 8, April 2020; Kistemaker, D.A., et al., Control of position and movement is simplified by combined muscle spindle and golgi tendon organ feedback. Journal of Neurophysiology, 109(4):1126–1139, February 2013].

These additional results and discussion have been included in the updated manuscript, along with modifications to the text and the incorporation of the new Figure 7 and Supplementary Figure S17:

Manuscript page 19, *Figure 7* and *caption:*

*"**Spino-cerebellar, SR-cerebellar, RI-cerebellar and cerebellar model responses to external motor perturbations during bell-shaped flexion-extension trajectories. A)** Kinematic performance of both the spino-cerebellar and cerebellar models under a forward perturbation at the flexed position whilst performing the 2.3s flexion-extension trajectory. 50 trials are displayed. **B)** Position deviation (ΔMAE) caused by all the perturbations applied during the 2.3s flexion-extension trajectory for the four models. Mean ΔMAE and standard deviation (std) of 50 trials are displayed. **C)** Mean position ΔMAE and std for all the perturbations applied to the flexion-extension trajectories performed at different speeds (3s, 2.3s, 1.5s). 50 perturbed trials were used for each perturbation type. **D)** Velocity deviation (ΔMAE) caused by all the perturbations applied during the 2.3s flexion-extension trajectory for the four models. Mean ΔMAE and std of 50 trials are displayed. **E)** Mean velocity ΔMAE and std for all the perturbations applied to the flexion-extension trajectories performed at different speeds (3s, 2.3s, 1.5s). 50 perturbed trials were used for each perturbation type. E) Shoulder and elbow CCI values for the four models. Mean and std of 50 trials without perturbation are displayed."*

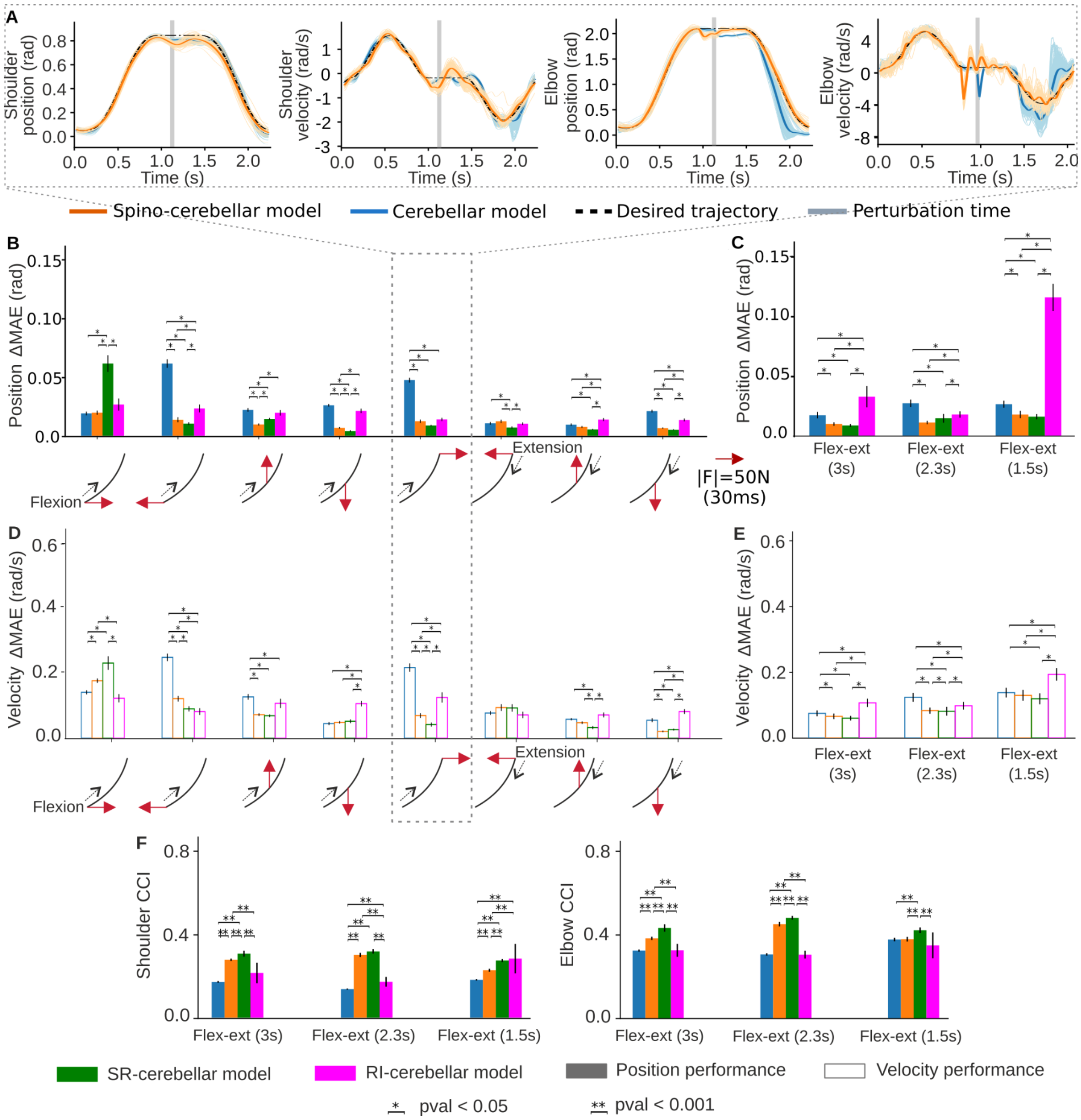

Manuscript lines 319-367, *Results* section *2.5 The spinal cord increases the robustness against motor perturbations:*

"*Both models faced 2000 consecutive trials of the flexion-extension movement performed at different speeds (3s, 2.3s, 1.5s); after motor adaptation, both models succeeded in performing the target kinematics (see Supporting Information (S16 Fig.)). Once both models adapted to perform the desired trajectories, we tested the contribution of the SC in handling motor perturbations. For that, we induced a set of external forces: i.e., 50 N for 30 ms applied to the hand in different directions and at different points along the flexion-extension movement, resulting in kinematic deviation (Fig. 7A). We then measured the MAE deviation from the ideal, no-perturbation scenario (Fig. 7B-E). Each perturbation type was applied on 50 separate trajectory trials to get an average response (see Methods). Besides, to gain a deeper understanding of the SC involvement in handling perturbations, we individually assessed the influence of the stretch reflex and reciprocal inhibition mechanisms. To that end, we applied the aforementioned set of external perturbations to two additional cases: i) SC equipped with just stretch reflex (SR-cerebellar model); ii) SC equipped with just reciprocal inhibition*

(RI-cerebellar model). In order to focus on the contribution of the two SC mechanisms and conduct a direct comparison between them, for the SR-cerebellar and RI-cerebellar cases, we set the cerebellar synaptic weights to those developed by the spino-cerebellar model. More specifically, we equipped the cerebellar network with the synaptic solution acquired by the cerebellum after motor adaptation when the SC model was fully equipped, thus preventing the cerebellum from learning how to compensate for the deficiencies induced by the lack of spinal mechanisms. The cerebellar learning capability was disabled *in all four cases* to prevent adaptation to the perturbations. *Note that, in previous research, we studied the cerebellar ability to effectively handle external perturbations during motor adaptation under changing task conditions [Luque et al., 2011, Abadía et al., 2019]. By disabling the cerebellar learning capability, we could specifically focus on the SC contribution.*

The kinematic performance of both the full spino-cerebellar and cerebellar models under one perturbation type, whilst performing the moderate flexion-extension movement (2.3s), shows that the cerebellar model exhibits a larger kinematic deviation compared to the spino-cerebellar model, particularly at the elbow level (Fig. 7A). When analysing the responses of the four models (spino-cerebellar, cerebellar, SR-cerebellar, and RI-cerebellar) to the set of perturbations applied during the moderate flexion-extension trajectory (Fig. 7B and D), we found that: i) the spino-cerebellar model exhibited significantly smaller Mean Absolute Error deviation (ΔMAE) compared to the cerebellar model, both in terms of position and velocity, for the majority of the applied perturbations; ii) the SR-cerebellar model exhibited smaller ΔMAE compared to the RI-cerebellar model. Additionally, the SR-cerebellar and spino-cerebellar models exhibited similar deviations for most of the perturbations. Similar results were obtained for the slow and fast bell-shaped flexion-extension trajectories (please refer to Supporting Information for the corresponding figure, S17 Fig.).

The kinematic performance analysis on the deviations of the four models in response to all the applied perturbations during the different trajectories (Fig. 7C and E), confirmed the similar behaviour of the spino-cerebellar and SR-cerebellar models. Both models exhibited superior performance and greater robustness compared to the cerebellar and RI-cerebellar models. All these findings support the role of the SC in handling external motor perturbations, and suggest that the stretch reflex component plays a dominant and more effective role in dealing with perturbations, whilst the reciprocal inhibition mechanism is not as extensively involved.

Finally, we computed the joint CCI values of our four models (Fig. 7F) during the three trajectories without perturbations. This analysis aimed to determine whether the CCI could serve as a biological marker to predict the performance against perturbations. The results showed that the SR-cerebellar model exhibited the highest CCI values, followed by the spino-cerebellar model, the RI-cerebellar model, and lastly the cerebellar model. This observation indicates a correlation between higher CCI values and greater robustness against perturbations, as models with higher CCI values exhibited smaller trajectory deviations. Based on these findings, we can conclude that the presence of the spino-cerebellar pathway contributes to better handling external motor perturbations, with the stretch reflex playing a prominent role, which leads to increased cocontraction levels."

Manuscript lines 428-441, *Discussion section:*

"The CCI increment was also revealed when inducing perturbations in the control loop; the spino-cerebellar model provided a better response, reducing the kinematic deviation. Further analysis of the SC mechanisms revealed the stretch reflex to provide better responses to perturbations than the reciprocal inhibition mechanism (SR-cerebellar vs. RI-cerebellar models), showing smaller kinematic deviation whilst also exhibiting higher joint CCI values. Thus, the stretch reflex was found to be a dominant mechanism in the SC improvement of kinematic performance under external perturbations,

whilst the inhibitory action of the reciprocal inhibition was not as extensively involved. Muscle elasticity has been previously identified as a significant passive contributor in handling perturbations [Burdet, E., et al., (2005)]. Within our framework, the spino-cerebellar and cerebellar models, as well as the SR-cerebellar and RI-cerebellar models, all used identical muscle models with the same mechanical properties. Consequently, the passive muscle contribution to handling perturbations remained equal in all cases. This direct equivalence allowed us to focus on the contribution of the SC and attribute a pivotal role to the SC through the stretch reflex in providing robustness against external perturbations, thus supporting previous physiological and modelling research [Shemmell, J., et al., (2010); Weiler, J., et al., (2019); Stollenmaier, K., et al., (2020); Kistemaker, D.A., et al., (2013)]."

Manuscript lines 814-820, *Methods* section *4.6.4 Measuring robustness against perturbations:*

"*We also computed the standard deviation. The MAE deviation results of the four models (spino-cerebellar, cerebellar, SR-cerebellar, and RI-cerebellar) were compared using a Kruskal-Wallis H-test [Kruskal, W. H., & Wallis, W. A. (1952). Use of ranks in one-criterion variance analysis. Journal of the American Statistical Association, 47(260), 583-621.] (computed using Python function scipy.stats.kruskal [Virtanen, P., et al., (2020). SciPy 1.0: fundamental algorithms for scientific computing in Python. Nature methods, 17(3), 261-272.]) to assess the overall difference amongst the four cases, followed by a Dunn test [Dunn, O. J. (1964). Multiple comparisons using rank sums. Technometrics, 6(3), 241-252.] (computed using Python function scikit-posthocs.posthoc_dunn [Terpilowski, M. A. (2019). scikit-posthocs: Pairwise multiple comparison tests in Python. Journal of Open Source Software, 4(36), 1169.]) to conduct pairwise tests. We also compared the mean joint CCI values of the four models during 50 trials for each of the three trajectories, performed without perturbations.*"

We thank the reviewer again for the valuable comments and hope that our efforts have addressed the raised concerns.

New *Discussion subsection* ***Cerebellum and spinal cord modelling assumptions.*** Manuscript lines 477-560.

3.1 Cerebellum and spinal cord modelling assumptions

The implemented cerebellar and spinal cord computational models are physiologically based and adhere to the principle of biological plausibility. Nonetheless, certain assumptions were made to facilitate interpretation of the results.

Regarding the cerebellum, it is well-known for its role in motor adaptation and learning, a role mainly supported by the cerebellar ability to acquire internal models of both body-plant dynamics and external objects [*Wolpert, D. M., et al. (1998)*]. These internal models are found to be either forward or inverse; a forward model maps the sensorimotor state to its predicted motor behaviour, whilst an inverse model maps the desired behaviour to the motor commands that will make it possible. The literature addresses the existence of both inverse and forward models in the cerebellum and, rather than confronting the two model alternatives, there is theoretical, computational, and behavioural experimentation that supports the coexistence and complementarity of both approaches [*Wolpert, D. M., et al., (1998); Ito, M. (2013); Honda, T., et al., (2018); Luque, N. R., et al., (2011); Passot, J. B., et al., (2013)*]. When modelled as a forward model, the cerebellum modulates the descending motor commands from the motor cortex to correct the mismatch between predicted and actual motor behaviour. In such cases, the body-plant dynamics are learnt at higher brain areas, and the cerebellum operates as a corrective mechanism in addition to the already learnt dynamics. Conversely, when functioning as an inverse model, the cerebellum does not rely on descending motor commands. Instead it can directly provide the entire motor output and bypass the motor cortex [*Ito, M. (1997)*]. In our work, the cerebellum is implemented as an inverse model, therefore putting it in a worst-case scenario in which it needs to learn the entire body-plant dynamics. The purpose of this approach is to directly address the influence that the SC has on cerebellar motor learning.

If the implemented cerebellar model were a forward model, it might improve tracking performance; however, it would hinder the evaluation of the influence of the SC on cerebellar learning. The SC has a direct and fast action over muscles, thus leading to significant modifications of the arm-plant dynamics. The SC modification of the arm-plant dynamics would affect the cerebellar learning whilst also influencing the motor cortex output. If the SC control mechanisms were already being accounted for at higher brain areas, it would indeed pose challenges in evaluating and quantifying the SC effect on cerebellar learning. It would be difficult to compare the impact of the SC between our spino-cerebellar and cerebellar cases due to the potential overlapping factors incorporated by the already accounted SC control mechanisms at higher brain areas. Using the cerebellum as an inverse model provides a direct means to assess the effects of the SC on cerebellar motor learning. By isolating the role of the cerebellum in motor control, we were able to study how the SC influences the cerebellar motor learning, i.e., comparison between our spino-cerebellar and cerebellar cases is straightforward.

To further facilitate the comparison between our spino-cerebellar and cerebellar cases, we simplified the action of the cerebellar output commands. In our cerebellar case, motor behaviour is solely driven by the cerebellum as the nervous region present in the control loop. Therefore, in this case, the cerebellar output commands directly controlled muscle activation. In the spino-cerebellar case, we maintained the cerebellar descending commands to the SC as direct control signals, which acted upon motoneurons, thus ensuring equivalent cerebellar output commands in both the

spino-cerebellar and cerebellar cases and facilitating directly studying the influence of the SC on cerebellar motor learning.

Regarding the cerebellar sensory input, it has been previously stated that within the dorsal spinocerebellar tract (DSCT) there is a subpopulation of neurons representing whole limb parameters and an equally-sized subpopulation representing joint-related signals. This halved structure allows for an early processing stage of sensory information that facilitates a reference frame for whole limb kinematics [*Bosco, G., & Poppele, R. E. (2001)*]. In our work, the focus was not on this early sensory processing in the DSCT. Instead, the proprioception signals provided by the SC in joint space were directly sent to the cerebellum. Nonetheless, despite its joint-based architecture, the cerebellum did account for whole limb kinematics as the driving force behind the motor adaptation process.

The structure of our cerebellar model involves assigning a cerebellar microcomplex to each joint, following Ito's microcomplex theory [*Ito, M. (1997)*]. Whilst the input sensory signals and output motor signals are joint-based, i.e., divided in a modular organisation of different cerebellar functional units [*Apps, R., et al., (2018)*], the cerebellar learning at GC - PC synapses, in contrast, considers the entire limb kinematics. GCs are believed to perform a recoding of sensory inputs [*Carrillo, R. R., et al., (2018); Cayco-Gajic, N. A., et al., (2017)*], ensuring an univocal and unambiguous representation of the kinematic state of the limb. GCs, in turn, transmit this sensory state to PCs which, driven by the CFs instructive action, adapt their activity to cope with the desired motor behaviour. Importantly, PCs are innervated by a massive number of GCs, with an estimated ~175,000 GC-PC synapses in the rat cerebellar cortex for each PC [*Napper, R. M. A., & Harvey, R. J. (1988)*]. Amongst that massive GC-PC innervation, PCs receive excitatory inputs not only from GCs within the very same microcomplex but also from GCs belonging to other microcomplexes. This connectivity allows the linkage of different cerebellar microcomplexes through GC-PC synapses [*Valera, A. M., et al., (2016); Apps, R., et al., (2018).*]. In our model, each PC receives excitatory input from all GCs, not just from those associated with the very same microcomplex. Therefore, each PC receives the entire sensory information from GCs, and a specific teaching/instructive signal through a particular CF corresponding to the same microcomplex, in accordance to CF-PC one-to-one synapses [*Eccles, J. C., et al., (1966)*]. This connection arrangement makes synaptic plasticity at PCs to be affected by the sensory information from all joints and enables the cerebellum to acquire a comprehensive internal model representation of the upper-limb, rather than a joint-specific representation. Therefore, despite the microcomplex structure being based on individual joints, the ensemble activity of GCs across PCs allows for motor adaptation to account for the entire limb kinematics.

Regarding modelling of the SC circuits, some assumptions were also made. The synaptic weights of the spinal pathways were selected aiming to reproduce physiological connectivity by maintaining the ratio between the amplitudes of excitatory postsynaptic potentials (EPSPs) from Group la afferents reported in [*Fleshman, James W., et al., (1981)*] and inhibitory postsynaptic potentials (IPSPs) from Group la inhibitory interneurons observed in [*Stuart, G. J., and S. J. Redman. (1990)*].

The modelled SC did not include heteronymous excitatory projections for the sake of interpretability. Heteronymous projections in the human upper limb have been effectively reported in [*McClelland, V. M., et al., (2001)*]. According to this study, inhibition occurs significantly more frequently than excitation between antagonist muscle pairs, with a higher frequency of inhibition received from antagonists muscles compared to muscles acting across a different joint. In [*Pierrot-Deseilligny, E., & Burke, D. (2012); Cavallari, P., & Katz, R. (1989*] it was noted that proximal to distal heteronymous Ia connections (from the arm to forearm) were absent, whilst wide connections

from distal to proximal muscles were present, although weaker from proximal to shoulder muscles. The authors suggested that these stronger connections at wrist and elbow level may assist the hand muscles in grasping and lifting movements by providing stability to the corresponding joints. Consequently, heteronymous excitatory projections are reported to have less significance than heteronymous inhibitory projections in human upper limb movements. These findings are aligned with our SC model, in which reciprocal inhibition pathways allowed to capture this behaviour.
