# Supplementary material for: The spinal cord facilitates cerebellar upper limb motor learning and control; inputs from neuromusculoskeletal simulation
Source: PLoS Comput Biol. 2024 Jan 2;20(1):e1011008. doi: 10.1371/journal.pcbi.1011008 (PMC10786408; doi:10.1371/journal.pcbi.1011008)
Supplement: Attachment — Submitted filename: response_reviewer.pdf [file pcbi.1011008.s021.pdf]

The authors would like to thank the reviewers and editors for their time spent on reviewing this work and their comments towards improving the manuscript. The comments and suggestions from the reviewers have been taken under consideration and have been addressed in the new version of the manuscript. Below, the authors have tried to answer the questions and reviewers' comments. Modifications within the manuscript have clearly been tracked in blue for an easy identification.

Reviewer #1: The authors have substantially revised the Ms and generated detailed responses to the reviewers.

- This model is based on Ito's 1997 presumption that an internal inverse model of the musculoskeletal plant can compute the motor commands required to achieve a desired behavioral goal. As recently pointed out, the oculomotor system for which this concept was derived is fundamentally different from most of the rest of the musculoskeletal system (Loeb, G. E., 2021, Learning to use Muscles, Journal of Human Kinetics, 76, 9). To their credit, the authors have included a small subset of the multiarticular muscles and dynamics, proprioceptive afferents and spinal circuitry that constitute this difference, but it remains unclear if the simplistic concept of joint-based microcomplexes will survive the larger reality. That reality is substantially underestimated by claiming that the spinal cord represents a "modification of the arm plant dynamics" (lines 499-500), particularly when considering that most of the cerebellar and cortical output is mixed with segmental sensory feedback in spinal interneurons before arriving at motoneurons. The authors' cerebellocentric view of sensorimotor control begs the question of where it interacts with the inevitable (but still unclear) contributions of the pyramidal cortical system. In addition to SC, a large part of that interaction is probably also in the reticular formation and deep cerebellar nuclei to which both project, a complication mostly ignored also by those who study and model cerebral cortex. A bit more discussion of these complexities in this already rather long paper might help cerebral and cerebellar researchers to stop talking past each other.

Answer

We want to thank the reviewer for helping us better clarifying the modelling assumptions and, thus, contextualise our work within the research in the field. While our models adhere to the principle of biological plausibility, we acknowledge that certain assumptions/simplifications were necessary to effectively address our primary scientific question: whether the spinal cord facilitates cerebellar motor learning.

Nonetheless, besides justifying the modelling assumptions in terms of the specific research being addressed, we concur with the reviewer that a clear depiction that links our models and the intricate reality of the nervous system is also essential. To address this, we have now expanded the "*Cerebellum and spinal cord modelling assumptions*" section of the manuscript. This extension now offers a more comprehensive discussion on how our models relate to the more complex reality of the nervous system, thereby providing readers with hopefully a clearer contextualisation within the broader scientific picture. Please, find the manuscript changes below. We thank the reviewer again for the efforts and time spent on reviewing our work, and we hope to have addressed the raised concerns.

Manuscript changes:

Line ~509:

To further facilitate the comparison between our spino-cerebellar and cerebellar cases, we simplified the action of the cerebellar output commands, by omitting other inputs to the spinal cord, e.g. from the motor cortex and basal ganglia. In our cerebellar case, motor behaviour is solely driven by the cerebellum as the only nervous region present in the control loop. Therefore, in this case, the cerebellar output commands directly controlled muscle activation. In the spino-cerebellar case, we maintained the cerebellar descending commands to the SC as direct control signals, which acted upon motoneurons, thus ensuring equivalent cerebellar output commands in both the spino-cerebellar and cerebellar cases. This simplified descending pathway facilitated directly studying the impact of the SC on cerebellar motor learning. Notably, our findings suggest that the SC facilitates cerebellar learning, as evidenced by the differences in the developed cerebellar solutions with and without SC involvement (i.e., spino-cerebellar vs. cerebellar cases exhibited different GC-PC synaptic weight distributions).

However, the intricate reality of the nervous system presents greater complexity; a significant convergence of cerebral and cerebellar efferent pathways occurs within the SC interneuronal circuitry before reaching motoneurons (Loeb, G. E., 2021, Learning to use Muscles, Journal of Human Kinetics, 76, 9). This raises a key question: do cerebellar internal models include this intermediary circuitry and account for the interaction amongst descending pathways, in addition to the dynamics of the musculoskeletal system?

Our work revealed that cerebellar learning is impacted by the interaction with SC circuitry, and therefore that spinal cord properties should not be underestimated when studying learning. Other descending pathways, such as those from the pyramidal cortical system, likely also impact cerebellar learning. Given that biological motor learning is determined by the interplay amongst various nervous regions, future research should expand this study to explore neural interactions that contribute to this intricate process.

Line ~536:

Our cerebellar model builds upon Ito's inverse model assumption [72], which derived from oculomotor system studies. Here, we extend that cerebellar scheme to the upper limb musculoskeletal system by including a subset of multi-articular muscles, with their associated dynamics, proprioceptive afferents and SC circuits. The structure of our cerebellar model involves assigning a cerebellar microcomplex to each joint, following Ito's microcomplex theory [72]. Whilst the input sensory signals and output motor signals are joint-based, i.e., divided in a modular organisation of different cerebellar functional units [74], the cerebellar learning at GC - PC synapses, in contrast, considers the entire limb kinematics.
